# Leader Cells Define Directionality of Trunk, but Not Cranial, Neural Crest Cell Migration

**DOI:** 10.1016/j.celrep.2016.04.067

**Published:** 2016-05-19

**Authors:** Jo Richardson, Anton Gauert, Luis Briones Montecinos, Lucía Fanlo, Zainalabdeen Mohmammed Alhashem, Rodrigo Assar, Elisa Marti, Alexandre Kabla, Steffen Härtel, Claudia Linker

**Affiliations:** 1 Randall Division of Cell & Molecular Biophysics, King’s College, London SE1 1UL, UK; 2 Laboratory for Scientific Image Analysis (SCIAN-Lab), Biomedical Neuroscience Institute (BNI), Centre for Medical Informatics and Telemedicine (CIMT), ICBM, Faculty of Medicine, University of Chile, Santiago 1058, Chile; 3 Instituto de Biologia Molecular de Barcelona, CSIC, Parc Cientific de Barcelona, C/Baldiri i Reixac 15-21, Barcelona 08028, Spain; 4 ICBM Human Genetics Program, Centre for Medical Informatics and Telemedicine (CIMT), Faculty of Medicine, University of Chile, Santiago 1058, Chile; 5 Engineering Department, Cambridge University, Trumpington Street, Cambridge CB2 1PZ, UK

## Abstract

Collective cell migration is fundamental for life and a hallmark of cancer. Neural crest (NC) cells migrate collectively, but the mechanisms governing this process remain controversial. Previous analyses in *Xenopus* indicate that cranial NC (CNC) cells are a homogeneous population relying on cell-cell interactions for directional migration, while chick embryo analyses suggest a heterogeneous population with leader cells instructing directionality. Our data in chick and zebrafish embryos show that CNC cells do not require leader cells for migration and all cells present similar migratory capacities. In contrast, laser ablation of trunk NC (TNC) cells shows that leader cells direct movement and cell-cell contacts are required for migration. Moreover, leader and follower identities are acquired before the initiation of migration and remain fixed thereafter. Thus, two distinct mechanisms establish the directionality of CNC cells and TNC cells. This implies the existence of multiple molecular mechanisms for collective cell migration.

## Highlights

• CNC rely on cell-cell interactions to migrate directionally
• Leader cells dictate directionality to followers in the trunk NC population
• Leader and follower identities are acquired before the initiation of migration
• Leader and follower identities are non-interchangeable during migration

## Graphical Abstract

**Figure:**
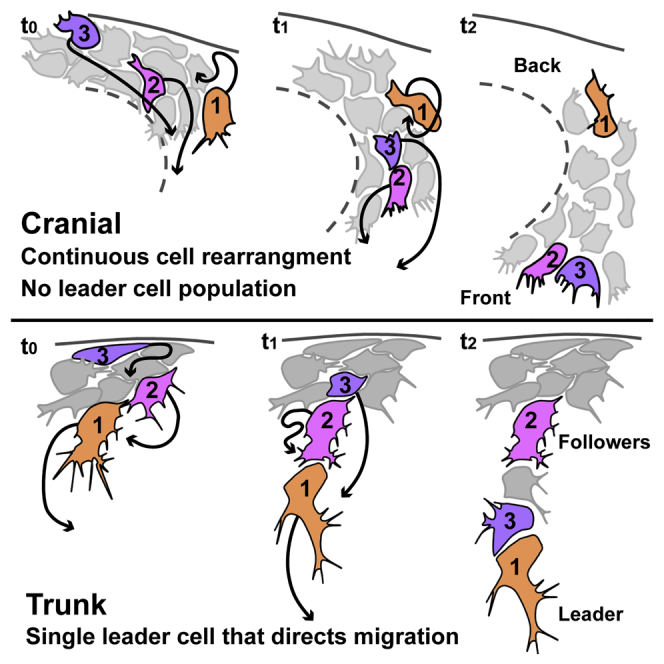

## Teaser

Richardson et al. uncover distinct migratory strategies used by cranial and trunk NC. Cranial crests are a homogeneous population in which directionality is acquired through cell-cell interaction. Trunk crests are heterogeneous at the onset of migration: leader cells dictate directionality and cell contact is required for migration.

## Introduction

Cell migration is fundamental for life, from organ formation to tissue repair and regeneration. Cells can migrate individually or collectively. Collective cell migration may endow cancer cells with an increased invasion capacity, which can result in aggressive tumor metastasis (Friedl et al., 2012). Cells migrating collectively maintain contact and read guidance cues cooperatively. These groups can adopt a range of spatial arrangements, from small numbers of loosely connected mesenchymal cells, to large masses of tightly associated cells (Friedl et al., 2012). Within these arrangements, cells may dynamically change position and rely on cell-cell interaction to determine directionality or be firmly positioned and play specific roles with leading cells directing movement (Mayor and Etienne-Manneville, 2016, Rørth, 2012).

Neural crest (NC) cells are a highly migratory embryonic population that shares many characteristics of metastatic cells (Maguire et al., 2015). Historically, NC cells have been described as cells that migrate individually (Le Douarin and Kalcheim, 1999), but recent work on chick and *Xenopus* embryos have demonstrated that cranial NC (CNC) cells migrate collectively. Experiments in *Xenopus* suggest that a combination of mechanisms imbue the group with polarity, cohesion, and overall directionality (contact inhibition of locomotion, co-attraction, collective chemotaxis, and interaction with surrounding tissues), leading to the proposition that all CNC cells are equally capable of taking the leader position, but it is the interaction between cells that endows the group with polarity and persistent migration (Scarpa and Mayor, 2016). By contrast, mathematical modeling and gene expression analyses of chick CNC cells have given rise to an alternative proposition, whereby cells adopt different identities depending on their position within the group. Leader cells, at the front of the group, are the only cells capable of directing migration, while trailers are guided by direct contact to a leader or to a trailer cell that has made contact with a leader (McLennan et al., 2012, McLennan et al., 2015a).

While CNC cells have been the subject of intense research, trunk NC (TNC) cells have attracted less attention. TNC cells migrate in two waves. First, they invade the space between the somites and the neural tube/notochord, named the medial pathway. Subsequently, TNC cells move between the ectoderm and the somites into the lateral pathway (Raible et al., 1992). Live imaging in chick has revealed that TNC cells migrating into the medial pathway do so in streams with close cell-cell interaction (Kasemeier-Kulesa et al., 2005, Krull et al., 1997). Moreover, video-microscopy analysis of zebrafish TNC cells has shown that NC-NC cell contact leads to collapse of membrane protrusions (Jesuthasan, 1996), similar to the mechanism of contact inhibition during CNC cell migration (Carmona-Fontaine et al., 2008). While these studies suggest that cell-cell interaction may also play a role during TNC cell migration, the topology, dynamics, and cellular regulation of migration remain largely unknown.

To better understand TNC cell migration and distinguish between the different models proposed to control CNC cell migration, we have conducted in vivo imaging and quantitative analysis in chick and zebrafish embryos. We found that all CNC cells present similar migratory behaviors and that leader cells are not a permanent population at the front of the group: instead, cells readily intermingle as they migrate, integrating into the leading edge only transiently. Moreover, laser ablation experiments in zebrafish embryos show that leader cells are not required for CNC cell directional migration. TNC cells, on the other hand, present a remarkably different migratory behavior. They move as single cell chains with division of labor: leader cells are permanently positioned at the front, instructing directionality to the entire group, while follower cells form the body of the chain and require cell-cell contact for migration. Leader and follower identities are defined before the initiation of migration and remain fixed thereafter. Our data show that TNC cells are a heterogeneous population at the outset of migration, consistent with a mechanism of fate restriction defining their migratory paths and behaviors (Raible and Eisen, 1994).

## Results

### CNC Cell Migration Does Not Require Leader Cells

**Figure 1:**
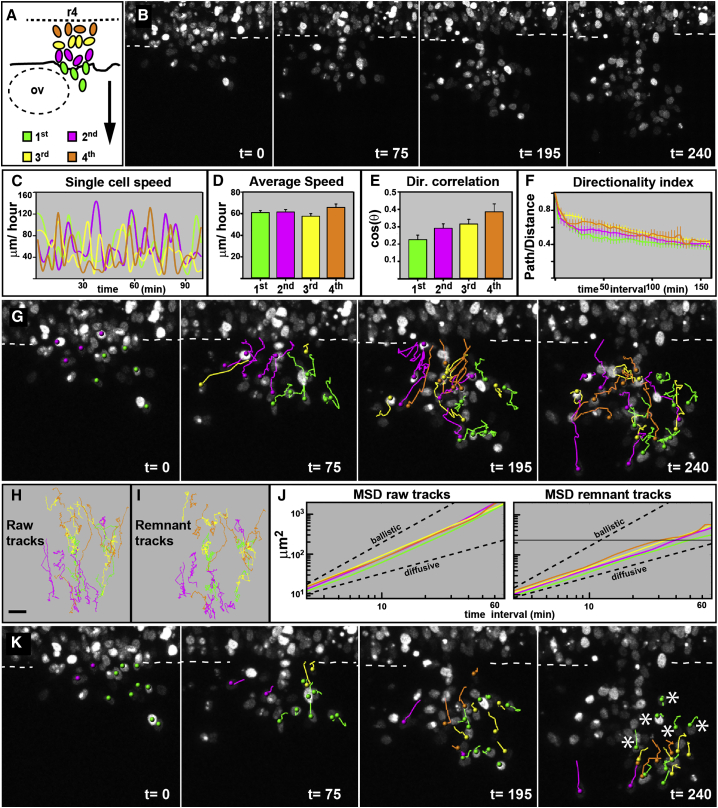
CNC Cells Are a Homogeneous Migratory Population Time in minutes. OV: otic vesicle. Dorsal views anterior to the left. Error bars represent SEM. (A) Diagram of CNC cells at the level of rhombomere IV. First, second, third, and fourth refers to the time at which cells initiate their migration. The arrow is a directional correlation vector. (B) Selected frames of Movie S1. Dotted line indicate the edge of the neural tube. (C) Speed of representative cells over time. (D) Average cell speed. (E) Directional correlation. (F) Directionality index. (G) Tracks of representative cells. (H and I) Raw (H) and remnant (I) tracks. (J) MSD of raw and remnant trajectories. The dashed lines show the ballistic and diffusive curves. The solid gray line marks the average cell size. X and Y have logarithmic scales (number of cells analyzed: first = 30, second = 30, third = 30, and fourth = 33 from 3 embryos). (K) Tracks of all cells that finalize their migration at the front of the group and cells of the first quartile. The asterisk mark cells that initiated migration at the front, but were left behind. See also Movie S1.

We set out to test whether CNC cells at different positions of the group have different or similar migratory capabilities. To this end, we performed live imaging of CNC cells at the level of the fourth rhombomere in chick embryos (Figures 1A and 1B) and developed computational tools to quantitatively analyze migration and morphology from these data sets in three dimensions. To compare the migratory parameters of cells at different positions, the group was subdivided in two different ways: (1) into quartiles according to their final location, corresponding to groups that have been shown to present distinct gene expression profiles (McLennan et al., 2015a) or (2) into quartiles according to the time at which cells initiate migration, which would set aside leader cells (Figure 1A). Independently of how the group was partitioned, no differences in speed or directionality of CNC cells were found (Figures 1C–1F). Thereafter, we used the time of initiation of migration to subdivide the group and analyzed how coherently cells move within the group. The two proposed models for NC cell migration generate different predictions: if the group is formed of cells with different identities, in which only leader cells are capable of directing migration, leader cells would present an advantage in retaining the front positions (McLennan et al., 2015a); alternatively, if all cells are equivalent and the group determines directionality through cell-cell interactions, the relative positions of the cells within the group would be irrelevant and cell intermixing would be observed. Consistent with data in *Xenopus* CNC cells (Carmona-Fontaine et al., 2008, Kuriyama et al., 2014), our analysis of cell trajectories shows that chick CNC cells readily intermingle as they migrate (Figures 1G and 1H; Movie S1). We used the mean square displacement (MSD) as a measurement of the area explored by cells (Gorelik and Gautreau, 2014) and found that all MSD curves present similar slopes, between the ballistic (fully directed movement, slope 2) and the diffusive (random walk, slope 1) slopes, as expected for directionally migrating cells (slopes first = 1.6, second = 1.7, third = 1.5, and fourth = 1.6 quartile; Figure 1J). To assess the relative movement of the cells within the group, we subtracted the average movement of each quartile (which accounts for common directional migration) from every cell trajectory and obtained the remnant movement (which accounts for the movement of cells with respect to each other). Remnant trajectories qualitatively show that all cells readily intermix (Figure 1I). Moreover, the remnant MSD curves present similar slopes that are close to the diffusive curve, indicating that cells move randomly with respect to each other (first = 1.1, second = 1.2, third = 1.2, and fourth = 1.2 quartile; Figure 1J). From these data, we can estimate that cells exchange neighbors every time they move more than one cell diameter (15 μm on average), or every 35 min. Next, we directly measured the rearrangements of CNC cells over time. Cells that initiate their migration as leaders are quickly left behind, and the front quartile is integrated by cells arising from all other quartiles (Figure 1G; Movie S1). Only 17.6% (3/17) of first quartile cells retain their leader position, while 77% (71/92) of all cells integrate a different subpopulation during the course of the experiment (e.g., first to second quartile or any other permutation). On average, neighbors (any pair of adjacently moving nuclei) migrate together for 37 ± 13.7 min (mean ± SD), while cells retain the leader position for 63 ± 49.2 min. Taken together, our data show that chick CNC cells present similar migratory parameters and do not maintain their relative positions as they migrate, suggesting that leader cells are not required to direct the movement of the group.

**Figure 2:**
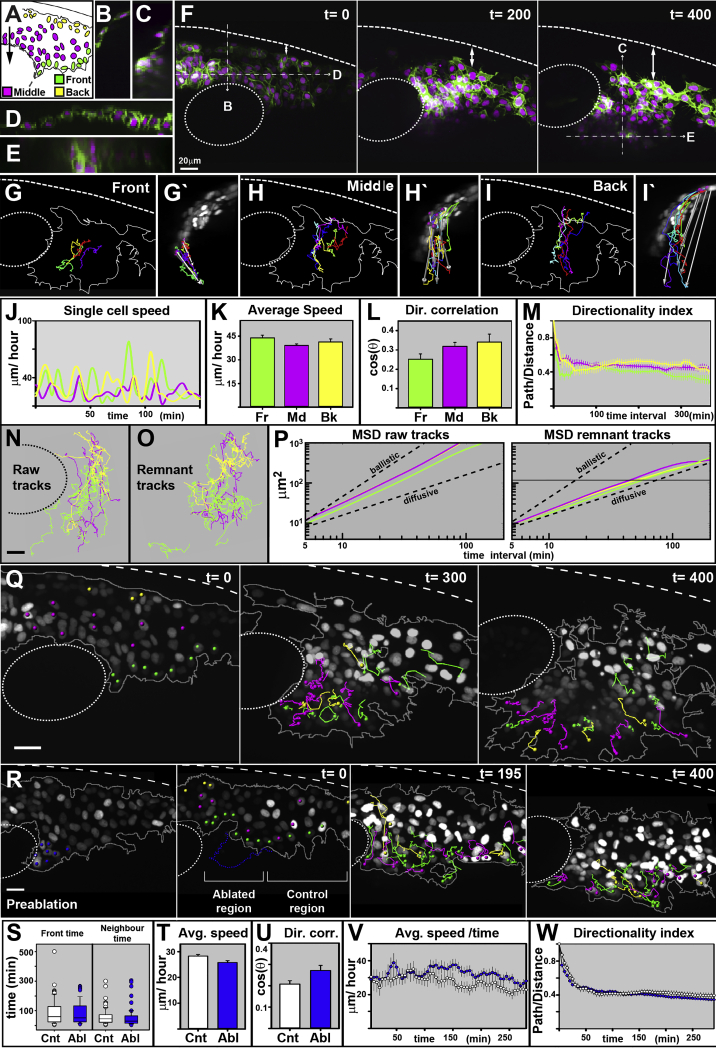
CNC Migration Does Not Require Leader Cells Time in minutes. OV: otic vesicle. Lateral views and anterior to the right. The error bars represent SEM. (A) Diagram of CNC cells anterior to the otic vesicle. The arrow shows the directional correlation vector. (B–E) Transverse (B and C) and coronal sections (D and E) indicated in (F). (F) Selected frames of Movie S2. The double arrow shows the growing dorsal area void of CNC cells. (G–I’) Tracks of representative front (Fr; G and G’), middle (Md; H and H’), and back (Bk; I and I’) cells. Lateral (G–I’) and transversal (G’, H’, and I’) view. The grey arrows connect the initial to the final track point, showing the deviation produced by 2D tracking. (J) Speed of representative cells over time. (K) Average cell speed. (L) Directional correlation. (M) Directionality index. (N and O) Cell tracks of raw (N) and remnant (O) movement. (P) MSD of raw and remnant trajectories. The dashed lines show the ballistic and diffusive curves. The solid black line marks the average cell size. A logarithmic scale is used in X and Y. Front, Fr = 38, middle, Md = 39, and back, Bk = 24 cells were analyzed from 4 embryos. (Q) Tracks of cells that initiate migration at the front of the group (green) and cells that finalize migration at the front of the group. Selected frames of Movie S3. (R) Preablation frame. The ablated nuclei are marked in blue. Selected frames of Movie S4. The color green shows the nuclei at the front, the color magenta shows them at the middle, and the color yellow shows them at the back after ablation. The dashed blue line shows the position of themembrane before ablation. (S–W) A comparison between control (white) and ablated cells (blue) of time at the front of the group and of neighboring nuclei (S), average speed (T), directional correlation (U), average speed over time (V), and directionality ratio (W). A total of 30 and 33 cells from ablated and non-ablated regions, from 3 embryos, were analyzed. All scale bars = 20 μm. See also Movies S2, S3, and S4.

Next, we set out to test the requirement of leader cells for CNC cell migration in zebrafish embryos. This animal model is particularly advantageous due to its genetic tractability and optical transparency, which permits high resolution live imaging concomitant with targeted cell ablations. We generated a new zebrafish transgenic line in which all NC cells have their nuclei and membranes fluorescently labeled (Sox10:mG; Figure S1) and quantitatively analyzed migration and morphology in vivo and in three dimensions. First, we studied the migratory behavior of pre- and postotic CNC cells. Consistent with previous work (Eisen and Weston, 1993), we found that zebrafish CNC cells arise as a monolayer at the dorsal region of the neural tube and migrate ventrally developing a multilayered structure (Figures 2A–2F; Movie S2). The zebrafish CNC cell group was subdivided into three subpopulations (Figure 2A): front cells (Fr) that present membrane to the leading edge of the group (Figures 2G and 2G’); middle cells (Md), which are surrounded by CNC cells (Figures 2H and 2H’); and back cells (Bk) that expose membrane to the rear of the group (Figures 2I and 2I’). The behavior of zebrafish CNC cells was very comparable to that of chick CNC cells: all CNC cells presented similar speed, temporal, and spatial directionality (Figures 2J–2M). The analysis of the cell trajectories from the three populations showed that cells readily intermingle as they move (Figure 2N), and their MSD curves present similar slopes, between the ballistic and diffusive movement (slope Fr = 1.4, Md = 1.5, and Bk = 1.6; Figure 2P). These dropped toward the diffusive slope when the directional movement of the group was subtracted (Fr: 1.1, Md: 1.0, and Bk: 1.0; Figures 2O and 2P), indicating that, as in the chick, zebrafish CNC cells move randomly with respect to each other. From these data, we can estimate that cells exchange neighbors every time they move more than one cell diameter (12.5 μm on average) every 50 min.

Next, we directly measured the temporal dynamics of cell rearrangements. Cells that initiate their movement at the front of the group are quickly left behind and replaced by cells arising not only from the entire span of the middle subpopulation, but also from those that initiate their migration at the back of the group (Figure 1Q; Movie S3). Only 5.3% (2/38) of front retain their leader position, while 72% (72/101) of all cells integrate a different subpopulation during the course of the experiment (e.g., back to middle). On average, cells remain neighbors for 57 ± 53.7 min, while cells maintain the front position for 78.4 ± 67.6 min (mean and SD; Figure 2S). These data corroborate our observations in chick CNC cells showing that, independently of their position, zebrafish CNC cells present similar migratory parameters, readily intermingle as they migrate, and do not present a resident leader cell population, suggesting that all the cells of the group have similar migratory capabilities and leader cells are not required for directional migration.

To test this hypothesis directly, we laser ablated leader cells and monitored the migration of the remaining cells. Two types of ablation were performed; either the first row of cells at the leading edge, or the first quartile of the group (approximately the first three rows of cells). In both cases, the migration of the remaining cells was unaffected (Figures 2Q and 2R; Movie S4): average speed and directionality showed no differences between ablated and control cases (Figures 2T and 2U). We reasoned that if leader cells were required for migration, but rapidly replaced, transient changes in the speed or directionality of the remaining cells may be obscured in the average calculations. Hence, we studied the behavior of cells over time, but again found no differences between the control and ablated cases (Figures 2V and 2W). Finally, we did not detect changes in the temporal dynamics of cell rearrangements as a consequence of the ablation procedure (Figure 2S).

Taken together, our data of chick and zebrafish embryos demonstrate that all CNC cells present similar migratory parameters, undergo extensive and constant rearrangements, and that leader cells are not required for their collective migration. We conclude that all cells in the group present equivalent migratory capacities.

### TNC Cells Are Composed of Three Populations: Leader, Follower, and Premigratory Cells

**Figure 3:**
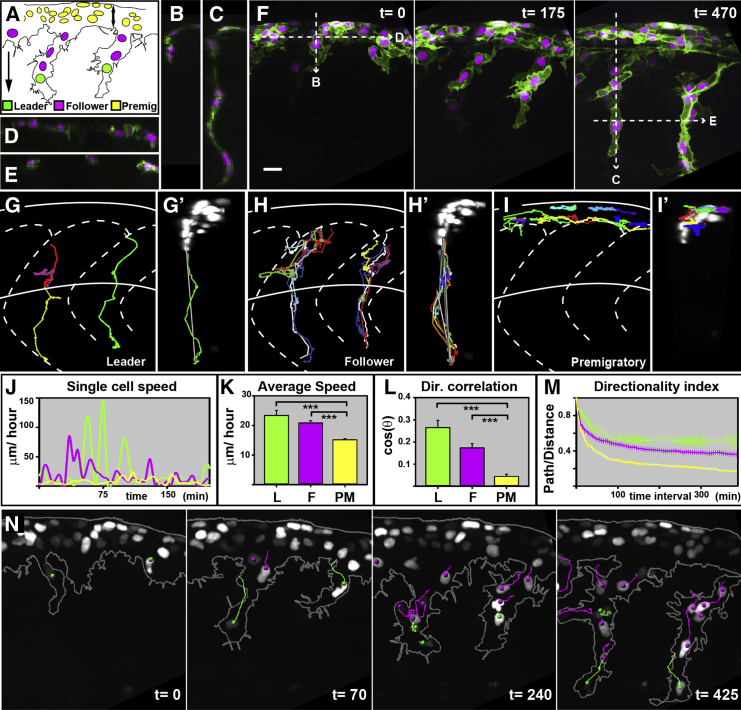
TNC Are Formed of Three Different Cell Populations Time in minutes. Lateral view and anterior to the left. The error bars represent SEM. (A) Diagram of migrating TNC cell leader, L, green; follower, F, magenta; and premigratory, PM, yellow. The double arrow marks the premigratory area. The simple arrow shows the directional correlation vector. (B–E) Transversal (B and C) and coronal (D and E) optical sections indicated in (F). (F) Representative frames of migrating TNC cell (Movie S5). (G–I’) Tracks of representative L (G and G’), F (H and H’), and PM (I and I’) cells. Lateral (G, H, and I) and transversal (G’, H’, and I’) views. (J) Speed of representative cells over time. (K) Average cell speed. (L) Directional correlation. (M) Directionality index (L = 15, F = 83, and PM = 85 cells, from 6 embryos, were analyzed). (N) Representative frames of migrating TNC cell (Movie S8) showing the tracks of L and F cells. All scale bars = 20 μm, valid for all panels. See also Movies S5, S6, S8, and S9.

We next turned our attention to the migratory behavior of TNC cells in zebrafish embryos. Similar to CNC cells, TNC cells arise as a monolayer at the dorsal region of the neural tube, but thereafter the migratory behavior of these populations differs. TNC cells do not move as a cohesive group; instead, a pool of motile cells remains dorsally in the premigratory area (Figures 3A, 3I, and 3I’). These cells occupy a constant region extending 26.1 ± 3.1 μm ventrally from the top of the embryo and contain on average 11.3 ± 3 cells per segment at any given time (average ± SD; consistent with Raible et al. 1992). From the premigratory area, TNC cells migrate as single cell chains between the neural tube and the somite into the medial pathway (Figures 3A–3F). A single leader cell initiates the chain and is trailed with high accuracy by follower cells (Figures 3F–3H’). Follower cells form the body of the chain connecting leaders to premigratory cells through cell-cell contact (Figures 3C and 3F; Movie S5). High resolution in vivo imaging shows that these contacts are sustained but very dynamic. Consistent with previous studies (Jesuthasan, 1996), and similar to CNC cells, TNC cells protrusions collapse upon contact (Movie S6), suggesting contact inhibition of locomotion between TNC cells.

Next, we tracked the movement of TNC cells in 3D and quantified their migratory behavior. Single cell speed curves show that, as for CNC cells in chick and zebrafish, TNC cells move in a saltatory manner. Leader and follower cells show high, but infrequent, acceleration peaks, while premigratory cells present very minor fluctuations (Figure 3J). Leader and follower cells are significantly faster than premigratory cells (Figure 3K), but intriguingly all TNC cells are slower than CNC cells (compare to Figure 2K). Analysis of directionality also shows significant differences: leader and follower cells are temporally and spatially more persistent than premigratory cells (Figures 3L and 3M). Next, we studied whether cells retain their relative positions and how coherently the migratory chains move. Contrary to CNC cells, trunk leader cells retain their front position (27/30, the three cases of leader cell replacement are described below; Figure 3N). In contrast, follower cells actively rearrange as they move (Figure 3N; Movie S5), leading to overtaking events that maintain the single cell topology of the chain (Figures 3C, 3E, and 3F).

**Figure 4:**
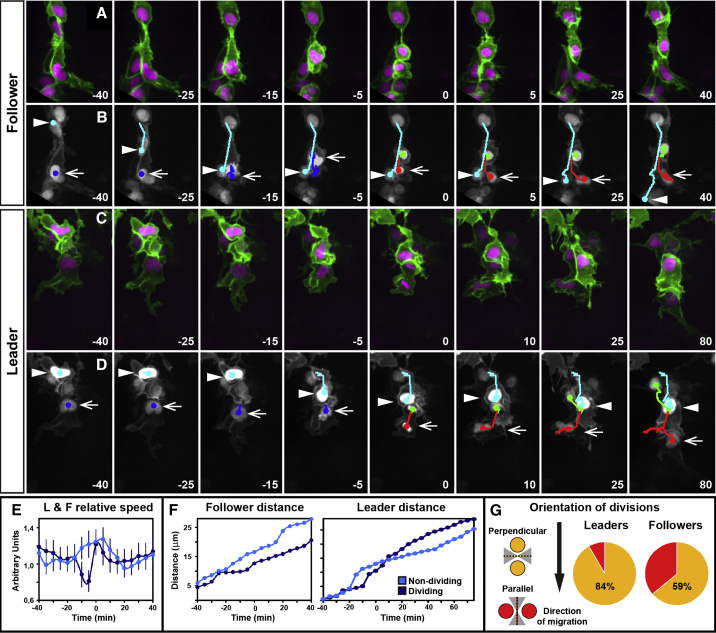
Trunk Leader and Follower Cells Present Different Division Dynamics L = 12 and F = 36 from 6 embryos, were analyzed Time in minutes and t_0_ = first frame with two separated nuclei. Lateral view and anterior to the left. The error bars represent SEM. (A and B) Selected frames of a dividing follower cell (Movie S7) (B) and its nuclear tracks, arrow points to dividing follower cell from −40 to 0 thereafter to its front daughter. The arrowhead points to a non-dividing neighbor. (C and D) Selected frames of a dividing leader cell (Movie S7) and (D) its nuclear tracks, the arrow marks dividing leader from −40 to 0 thereafter to its front daughter. The arrowhead marks a non-dividing neighbor. (E) Average speed ratio (speed at t_n_/average speed), error bars represent SEM. 17 dividing and 20 non-dividing cells, from six embryos, were analyzed. (F) Left, cumulative distance covered by a representative dividing follower and its non-dividing neighbor. The cumulative distance covered by a representative dividing-leader and its non-dividing follower is shown on the right. (G) Planes of division categorized as parallel (red) or perpendicular (yellow) relative to the direction of migration (arrow) within 45 degrees (gray shade). See also Movie S7.

Next, we turned our attention to the proliferative behavior of TNC cells. Previous studies in chick and *Xenopus* have shown that dividing CNC cells remain motile during division (Carmona-Fontaine et al., 2008, Ridenour et al., 2014). In contrast, our data show that migrating TNC cells stall their movement before division, regaining speed after cytokinesis (Figures 4A–4E; Movie S7). As a consequence, dividing follower cells cover shorter distances (Figure 4F) and are often overtaken by non-dividing neighbors (19/30; Figures 4A and 4B). Interestingly, while leader cells also stall movement before cytokinesis (96.25 ± 83.09 min before division, average and SD), they are not overtaken by follower cells (16/19; Figures 4C–4E; Movie S8). We found three exceptions where leaders that stall movement for exceptionally long periods before division (285, 305, and 435 min) were overtaken. Surprisingly, in all three cases, the new leader cells originated from the premigratory area and not from the followers pool (Movie S9). This suggests that leaders’ arrest is communicated to premigratory cells, which are the only source of new leader cells.

Finally, we analyzed the orientation of leader cell cytokinesis, which preferentially divide perpendicular to the direction of migration (16/19; Figure 4H). While leaders’ daughters present similar sizes after cytokinesis (data not shown) they differ in their behavior. The front daughter cell becomes the new leader and the back one a follower cell (16/19 cases; Movie S7). Follower cells, however, do not show such bias (Figure 4H).

In conclusion, TNC cells present three different cell populations with distinct migratory behaviors: leader cells are a permanent population at the front of the group that moves faster and more persistently; follower cells trail leaders and intermix as they migrate; and premigratory cells remain in the dorsal-most region of the embryo.

### TNC Leader Cells Define the Directionality of Migration

**Figure 5:**
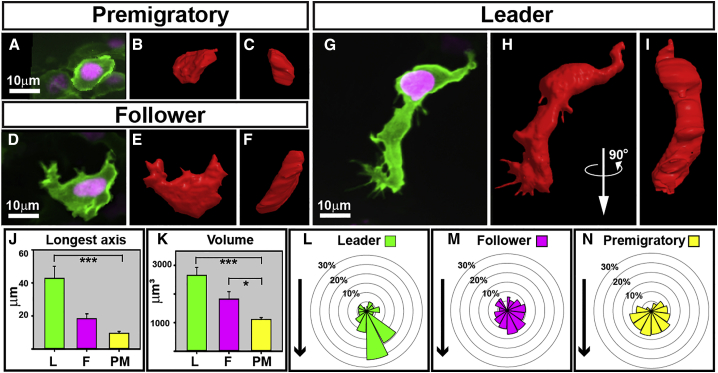
TNC Cells Show Different Morphology (A–I) 3D models of PM (A–C), F (D–F), and L (G–I) cells. Picture of the modeled cell (A–I), lateral (B, E, and H), and 90° rotation (C, F, and I) of the 3D model. (J and K) Mean longest axes and (K) mean volumes of L (n = 7), F (n = 10), and PM (n = 9), error bars represent SEM. (L–N) Angle of protrusions of L (n = 12, 217 protrusion), F (n = 24, 308 protrusion), and PM (n = 9, 141 protrusions) cells. The arrow shows the direction used to orient cells. See also Movie S10.

We next analyzed the morphology of TNC cells using 3D reconstructions from high resolution images (Figures 5A–5I; Movie S10). Interestingly, leader cells are the longest (primary axis: leader = 42.8 ± 2.6, follower = 18.5 ± 2.7, and premigratory = 9.6 ± 1 μm, average and SE), the largest (volume: leader = 2,653.8 ± 98.4, follower = 1,823.8 ± 78.2, and premigratory = 1,116.1 ± 19.6 μm^3^), and the only cells polarized in the direction of migration (Figures 5J–5N). Next, we asked whether these morphological differences are established before the initiation of migration or acquired during migration. We retrospectively tracked cells to the premigratory region and measured their area before the initiation of migration (Figure S2). Cells that divided within 90 min of the initiation of migration were not taken into account for this analysis, as these are expected to present large sizes. Surprisingly, prospective leaders presented larger areas (>175 μm), and by extension even larger volumes, than follower cells. These results suggest that TNC cell leader and follower identities are established at some point before the initiation of migration.

Next, we analyzed the size and behavior of cells that remain in the premigratory area after the leader’s departure. Throughout the course of the experiment, the premigratory area is formed of large and small cells in constant proportions (45% and 55%, respectively). Independently of their size, 50% of premigratory cells migrate as followers all showing similar migratory parameters (data not shown). Large premigratory cells divide more frequently than small ones (34% of large and 12% of small cells) and 16% of premigratory cells neither divide nor migrate, but remain resident in the premigratory area (Figure S2).

Leader cells are a permanent population at the front of the group formed of faster, more persistent, larger, and polarized cells. These traits suggest that they may be directing migration. To determine whether leader and follower cells migrate independently or interact to establish directionality, we performed a directional correlation analysis, in which pairs of cells migrating in the same direction present a higher correlation index than independent pairs. We considered two possibilities: (1) all cells follow the leader and (2) cells only follow their immediate front neighbor (Figure S3). Surprisingly, we found that the direction of a follower cell at any given time resembles more the direction of its leader than the direction of its front neighbor, supporting the idea that leader cells instruct directionality to the group. To test this hypothesis directly, we performed laser ablations of single leader cells.

**Figure 6:**
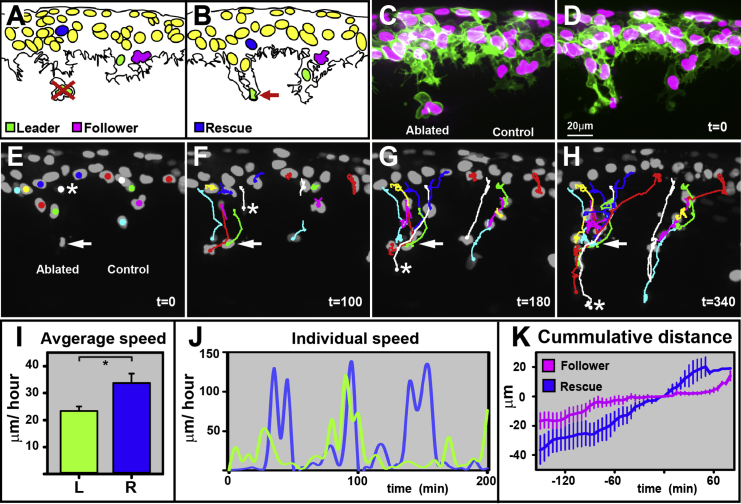
TNC Leader Cells Are Required for Migration Lateral view and anterior to the left. The error bars represent SEM. (A and B) Diagram of pre and postleader ablation. The red cross shows the targeted cell, and the red arrow shows the cell debris. (C and D) Pre and postablation snapshots. (E–H) Selected frames of a leader ablation movie, the second example in Movie S11. Asterisk mark rescuing cell. Arrow mark point of ablation. (I) Average speed of L (green, n = 15) and rescuing cells (blue, n = 4). (J) Speed of representative L and rescuing cells over time. (K) Cumulative distance covered by F (magenta, n = 4, and 3 embryos) and rescuing (blue, n = 5, and 5 embryos) cells (t_0_ time at which a cell overcomes the ablation point) ( 0 μm location of ablated cell) (total of eight experiments). See also Movie S11.

Upon leader ablation, the first follower cell actively protrudes into the newly available space, advancing to the ablation point, but it does not migrate further (Figures 6A–6E; Movie S11). Follower cells behind it behave similarly, remaining motile, but unable to migrate beyond the ablation point. As a consequence, the entire chain is blocked, with cells accumulating at the ablation site. Remarkably, migration is only reestablished once a cell that has not yet initiated its migration, located in the premigratory region at the time of ablation, moves to the front of the chain and takes on the leader’s role. The previously stalled follower cells then renew their directional movement by trailing the rescuing cell (Figures 6E–6H; Movie S11). Interestingly, rescuing cells are significantly larger than prospective follower cells before the initiation of migration (Figure S2), a feature that is shared with prospective leader cells.

Altogether, these data show that leader cells direct TNC cell migration, that follower and leader identities are acquired before the initiation of movement, and remain fixed thereafter.

### Cell-Cell Contact Is Required for TNC cell Migration

**Figure 7:**
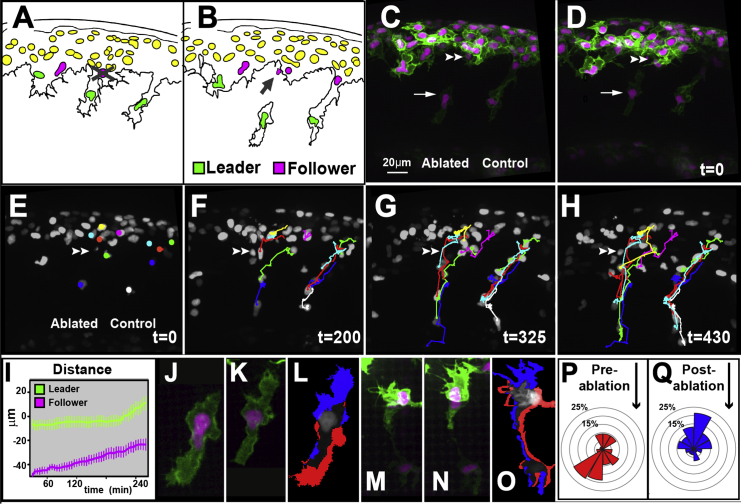
Cell-cell Contact Is Required for TNC Migration Lateral view and anterior to the left. The error bars represent SEM. (A and B) Diagram of pre- (A) and postgap (B) ablation. The gray cross shows the targeted cell, and the gray arrow shows the cell debris. (C and D) Pre- (C) and post- (D) ablation snapshots. The arrow points to the leader cell and the double arrowhead to the point of ablation. (E–H) Selected frames of a gap ablation movie, first example in Movie S12. (I) Cumulative distance covered by cells in front (green, n = 6, and 4 embryos) and behind (magenta, n = 4, and 4 embryos) the ablation (t_0_ time of ablation) (0 μm L location in the ablated chain) (total of seven experiments). (J–O) Enlargement of leader cell (J–L) or leader cell group (M–O) before and after a gap ablation. (L and O) Localization of the membrane extension (blue) and retraction (red) of the leader cells/group after the ablation. (P and Q) Quantification of the angle distribution of leader cells’ protrusions before (J, n = 4 cells, 54 protrusion, and 4 embryos) and after (K, n = 5 cells, 76 protrusion, and 5 embryos) ablation, the black arrow shows the direction to which all cells were oriented. See also Movie S12.

Next, we tested the role of follower cells during migration. First, we ablated an early migrating follower, thus severing the chain into two groups (gap ablation): the leader cell, alone or with a follower cell, and behind it, the remaining followers of the chain (Figures 7A–7D; Movie S12). These groups failed to migrate independently; the isolated leader cell (or group) repolarized backward and paused its movement, while follower cells advanced through the ablated region (Figures 6E–6M). The leader cell only resumed its movement once it had reestablished contact with the follower cells (Figure 7N; Movie S12). Next, we removed a late migrating follower maintaining cell-cell contact between the leader and the premigratory area (follower ablation, Movie S12). In this case, cells neighboring the ablation rapidly invaded the free space and the overall movement of the group was not affected.

Altogether these data show that direct cell-cell contact between the leader and followers is essential for the collective migration of TNC cells.

## Discussion

NC cells arise at the dorsal part of the embryo, from where they migrate extensively and colonize almost every tissue of the body. How such directed and organized migration is controlled remains an open question. Herein, we have quantitatively analyzed the migration of NC cells at different anteroposterior levels and addressed whether leader cells are required for directional movement. Examination of CNC cells in chick and zebrafish embryos shows that all cells in the group present similar migratory parameters and behavior. The group migrates directionally as a whole, but individual cells move randomly with respect to each other; consequently, cells only integrate the leading edge transiently. Moreover, laser ablation of leader cells does not affect the migration of the remaining group. From these data, we conclude that all CNC cells have similar migratory capabilities and that specialized leader cells are not required for directional migration. Analysis of TNC cells in zebrafish shows strikingly different behaviors. Leader cells are a permanent population at the front of the group with characteristic morphological and migratory parameters. Abrogation of the leader cell stalls the migration of followers, which remain motile, but are unable to progress ventrally. These experiments demonstrate that leader cells impose directionality to the group and that leader and follower identities are fixed and not interchangeable during migration.

### CNC Cell Directional Migration Is Achieved in the Absence of Leaders

CNC cells migrate as large cohesive groups forming streams. The collective migration of these cells has been recently established, but the mechanism governing this process remains controversial. Experiments in *Xenopus* embryos have led to the proposition that cell interactions confer polarity and persistent migration to the group (Mayor and Etienne-Manneville, 2016). An alternative model, based on experiments in chick CNC cells, postulates that leader cells at the front of the group direct movement, while trailer cells are guided by leaders through cell-cell contact. A gradient of vascular endothelial growth factor (VEGF) sculpted by CNC cells might provide a directional cue: leader cells would be able to bind and respond to this factor by moving forward, while follower cells would only bind to and consume VEGF, thus acting as a sink (McLennan et al., 2012). This model implies fundamental differences between leader and follower cells and is consistent with variations in the transcription levels of 70–90 target genes among CNC cells at different positions of the stream (McLennan et al., 2012, McLennan et al., 2015a). The first prediction arising from this model is that leader cells will preferentially retain the front positions during migration; leaders are the only cells capable of responding to VEGF and are permanently confronted by its highest concentrations in the gradient. Second, the group should migrate coherently with all cells orderly moving toward the VEGF gradient. Both of these predicted behaviors are observed in computational simulations of the model (McLennan et al., 2012), but not in vivo. In fact, long term video-microscopy of chick CNC cells show that neighboring cells can move in opposite directions (Kulesa and Fraser, 2000, Kulesa et al., 2000, Kulesa et al., 2008), while studies of *Xenopus* CNC cells demonstrate that cells do not maintain their relative positions and the group readily intermix during migration (Carmona-Fontaine et al., 2008, Kuriyama et al., 2014). Our quantitative analysis of CNC cell migration in chick and zebrafish embryos confirms these observations, showing that cells at the leading edge do not form a permanent population, but are constantly replaced, not only by cells immediately behind them, but also by cells that initiate their migration at the very back of the group. Cells randomly exchange positions as they move, and we consistently observed leader and follower cells migrating persistently against the flow of the group (Movie S1). Finally, the model requires leader cells for the directional migration of the group, as only leaders are capable of reading the directional cue. While the model allows followers to become leaders, such identity change requires gene transcription and should be accomplished in 45 to 60 min to maintain efficient migration (McLennan et al., 2015b). We experimentally tested these predictions by laser ablation of leaders (all cells at the leading edge or all cells in the first quartile) and found that CNC cell migration does not require leader cells. After laser ablation, follower cells positioned at the leading edge immediately repolarize and move ventrally without change in their speed, directionality, or dynamic rearrangements. These data show that follower cells replace leaders immediately (within 5 min), demonstrating that all cells of the group present equivalent migratory capabilities and suggesting that acquisition of leading edge characteristics is independent of changes in gene expression. These results are consistent with *Xenopus* data showing that all the cells of the group have the capacity of acquiring polarity and directional migration when presented to a CNC cell free area (Carmona-Fontaine et al., 2008). Moreover, they are in agreement with transplant experiments in chick embryos, where migration is not affected by the graft of trailing cells to the leading edge (McLennan et al., 2012).

We conclude that all CNC cells have equivalent migratory capacities and do not require specialized leader cells for migration. The transcriptional differences observed between leader and trailer CNC cells might be a consequence of unequal forces, topology, and interactions that cells at different positions of the stream sustain, but are unlikely to be the cause (or the signature) of specific migratory identities.

### TNC Cells Are Directed by Leader Cells

The migration of NC cells in the trunk region presents a very different topology than in the cranial area. TNC cells migrate as single cell chains extending from a large pool of motile cells that remain in the dorsal region. Our quantitative analysis shows that TNC cells are composed of three distinct cell subpopulations that play different roles during migration. Leader cells initiate the chain and retain the front position throughout the migratory process. These are larger and the only cells polarized in the direction of migration. They move fast and with sustained persistence. Interestingly, all the cells in the chain follow with more accuracy the leaders’ track than the path of cells in front of them. Together, these observations suggest that leaders orchestrate the movement of the entire group. Indeed, laser ablation of leader cells results in the arrest of ventral advance. Cells behind the leader remain motile, but are unable to acquire leaders’ traits and reestablish migration. The specificity of these results is confirmed by the fact that ablation of a single follower cell does not affect TNC cell migration. The ablation procedure and the number of targeted cells were similar in both cases; hence, the pause in migration following leader ablation is not caused by general tissue injury, death of surrounding non-fluorescent cells, or damage to more TNC cells than the targeted cell.

These data demonstrate leader cells are the only cells directing the migration of the group and suggest permanent molecular differences that distinguish leader from follower cells. Which factors may distinguish leader cell identity? In other contexts, leaders’ main functions are to remodel the substrate, read directional cues, and signal to the rest of the group (Khalil and Friedl, 2010, Rørth, 2012). These roles may be fulfilled by several molecular pathways that have been implicated in NC cell migration and further analysis will be required.

Follower cells trail leaders with great accuracy, connecting leaders to the premigratory area through cell-cell contact. This contact is dynamic and continuous, presenting the hallmark of contact inhibition of locomotion (CIL; Abercrombie, 1979), the collapse of protrusive activity at the point of contact. Moreover, when a gap is generated in the chain by ablation of a follower cell, cells repolarize against the direction of migration and movement is only restored once cell-cell contact is reestablished. This shows that cell contact is required for movement and that cell polarity is acquired as a consequence of contact. Significantly, some of the molecular players controlling CIL in CNC cells, such as *par3* and *N-cadherin*, are also present in TNC cells (Moore et al., 2013; J.R. and C.L., unpublished data), suggesting that CIL may play a similar role in the migration of CNC cells and TNC cells.

Follower cells undergo constant rearrangements, but maintain the single cell chain topology and are unable to overtake or play the leaders’ role upon ablation. Moreover, morphological differences between cells are observed before the initiation of migration. These results strongly suggest that leader and follower identities are defined before the onset of movement and remain fixed thereafter. How could these different identities be established? In other collective cell migration contexts such as angiogenesis, *Drosophila* trachea formation, and wound healing, intercellular competition mediated by the Notch pathway establishes leader cells (Affolter and Caussinus, 2008, Phng and Gerhardt, 2009, Riahi et al., 2015). Interestingly, Notch pathway components are expressed in TNC cells (Rios et al., 2011) and have been shown to participate in NC cell induction (Cornell and Eisen, 2005) and migration (De Bellard et al., 2002, High et al., 2007, Mead and Yutzey, 2012), raising the possibility that Notch signaling may be implicated in the selection of TNC cell identity. This process could also be influenced by communication between the migratory and premigratory cells, as suggested by the rapid migration of a premigratory cell to the front of the chain after leader cell ablation. It has been shown that gap junctions are required for NC cell migration (Huang et al., 1998a, Huang et al., 1998b, Waldo et al., 1999), and that NC cells exchange cytoplasmic material during migration (McKinney et al., 2011), providing a potential mechanism for rapid information flow through the chain into the premigratory region.

Independently of the molecular mechanisms defining premigratory cell identity, our results raise the matter of the time at which TNC cell identities are established. Premigratory cells may randomly initiate migration, sense their positions once part of the chain, and fix their identity thereafter; or cell identity may be predefined at some point before the onset of migration, with cells incorporating into the chains in order according to their identity. In either case once migration is initiated, premigratory cells can sense the state of the migrating chain and generate rescuing leader cells if required.

Interestingly, prospective and rescuing leader cells are larger than prospective follower cells when in the premigratory region. How can these differences in size be explained? Coordination of cell growth and cell-cycle progression occurs at the passage of G1 to S phase (Lloyd, 2013). It has been proposed that TNC cells only initiate migration as they enter the S phase (Burstyn-Cohen and Kalcheim, 2002). Our data raise the possibility that cell-cycle progression links cell size to migratory identity, controlling the onset of migration.

Once cells initiate movement they continually divide. Leader cells are biased to divide perpendicular to the direction of migration, with the front daughter retaining the leaders’ role. These observations suggest the asymmetrical distribution of leaders’ determinants upon division. While we could not observe morphological differences between the two leaders’ daughters, this is an interesting hypothesis that remains to be explored.

### Conclusions

Our results show that the migratory behavior of NC cells is different in the cranial and trunk regions, suggesting the existence of distinct molecular mechanisms controlling collective cell migration. These differences could be due to a combination of factors such as the intrinsic properties of NC cells and/or the spatial organization of the migrating group, but also some constraints imposed by the specific environment in which they migrate. In fact, CNC cells migrate between the neural tube and the epidermis, while medial TNC cells migrate between the somite and the neural tube/notochord. In the trunk, the presence of a leader cell may prevent cell intermingling at the front of the chain, facilitating the orderly movement of the cells along a relatively narrow path. In the head region, CNC cells migrate as a large compact group within a less constrained environment, which could allow the advance of the group with more freedom of individual cells within the population. Besides this, signaling cues may also play a role, such as differences in the composition of the extracellular matrix and the presence of distinct guidance molecules in the cranial and trunk regions. Identifying these differences and understanding how they impact on the migratory behavior of NC cells in vivo will provide critical insights into the molecular mechanisms of collective cell migration.

## Experimental Procedures

### Chick Time-Lapse Imaging

This study complies with all UK animal regulation and has been carried under the licenses and ethical approval required. Neural tubes of stage HH8-9 embryos were electroporated with Histone 2B-GFP plasmid DNA, EC cultures were performed and incubated for 8 hr before imaging, which was performed in a dorsal view, taking one image every 3′ during 10–12 hr in a ZEISS LSM780 system, 100 μm z stacks with 2.5 μm z-steps.

### Generation of the Sox10mG Transgenic Line

The 4.9 kb *Sox10* promoter (Carney et al., 2006) drives expression of the multicistronic open reading frame for H2B-monomeric Cherry (chromatin-label) and membrane tagged GFP (GPI), separated by the 2A viral peptide (Shioi et al., 2011). Cloning and transgenesis was performed according to the Tol2kit protocols.

### Zebrafish Time-Lapse Imaging and Laser Ablations

Somite 7–9 at 16 hpf (18–22 hpf for ablation experiments) were imaged laterally every 5′ for 16–18 hr using a PerkinElmer Ultraview Vox system. 70 μm z stacks with 2 μm z-steps were taken, except for membrane dynamics and 3D models, in which 1 μm z-step every 30” was used. A MicroPoint (Andor) laser was used for ablations. Damage to surrounding tissues was monitored with BODIPY TR methyl ester labeling (data not shown).

### Data Analysis

3D nuclear tracking was performed with the View5D ImageJ plugin, except for dividing cells, where center of mass was used. Directionality ratio d/D (persistence) measures the deviation between path distance (D) and linear distance from start to end point (d) (Gorelik and Gautreau, 2014). Directionality correlation compares the direction of a cell path to the ideal direction of migration. 3D models were generated with SCIAN-Lab software based on IDL 7.1.2 platform (Interactive Data Language, Exelisvis). Python and NumPy software were used for the MSD analysis. Angles of protrusions, plane of division, and cell area were measured manually with ImageJ. Student’s t test, Mann-Whitney, or Kruskal-Wallis tests were performed. Shapiro-Wilk test was used to compare directionality dynamics, and correlation tests were used to estimate linear correlations. Excel and SigmaPlot were used for statistical analysis and graphs.

For detailed protocols and Matlab scripts used see Supplemental Information.

## Author Contributions

J.R. performed the zebrafish experiments; L.F.E. and E.M. performed the chick experiments; A.G. designed and performed cell tracking and data analysis with L.B.M., Z.A., S.H., and A.K.; R.A. performed statistical analysis; and C.L. conceived and designed all experiments, prepared the figures, and wrote the manuscript.

## Supplemental Information

Document S1. Supplemental Experimental Procedures and Figures S1–S3

Movie S1. Cranial Neural Crest Migration in Chick Embryos, Related to Figure 1 The first panel shows a maximal projection of a preotic CNC cell time-lapse movie from a chick embryo in which the neural tube has been electroporated with H2B-GFP plasmid. This is followed by an overlay with the tracking of representative first, second, third, and fourth quartile separated by the time of migration initiation. The arrows point to cells that migrate against the overall directionality. The last panel shows the overlay of the tracking of the first cells that initiate migration, first quartile, and all other cells that finish their movement at the front of the group. The asterisks indicate cells that initiate migration at the front, but are left behind over the course of the experiment. The images were taken every 3 min, for a total duration of 237 minutes (dorsal view and anterior left).

Movie S2. Cranial Neural Crest Migration in Zebrafish Embryos, Related to Figure 2 The first panel shows a maximal projection of a postotic CNC cell time-lapse movie from a Sox10:mG embryo. This is followed by three panels of nuclear fluorescence projection overlaid by the tracking of representative front cells (presenting membrane to the leading edge), middle cells (surrounded by other NC cells), and back cells (presenting membrane to the rear of the group). The images were taken every 5 min, for a total duration of 400 min (lateral view, dorsal top, and anterior left).

Movie S3. Cranial Neural Crest Cell Intermixing during Migration in Zebrafish, Related to Figure 2 The first panel shows a maximal projection of a postotic CNC cell time-lapse movie from a Sox10:mG embryo. The second panel shows an overlay of the nuclear fluorescence and the track of cells that initiate their migration at the front of the group and all other cells that finish their movement at the front of the group. The third panel shows an overlay of the nuclear fluorescence and the track of cells that initiate their migration at the front of the group. The fourth panel shows an overlay of the nuclear fluorescence and the track of cells that initiate their migration at the middle of the group and finish at the front. The fifth panel shows an overlay of the nuclear fluorescence and the track of cells that initiate their migration at the back of the group and finish at the front. The images were taken every 5 min, for a total duration of 495 min (lateral view, dorsal top, and anterior left).

Movie S4. Cranial Neural Crest Leader Cell Ablation in Zebrafish, Related to Figure 2 The top panels on the right show a maximal projection of the fluorescent channels. The left panels show a nuclear fluorescence overlaid with the track of cells that were at the front of the group after the ablation procedure or that finished their migration at the front of the group. The first frame shows a preablation picture, and the blue indicates the nuclei that will be ablated. The second frame shows the initiation of the movie, and the blue dashed line indicates the membrane outline before the ablation procedure. The top panels show the ablation of the first row of front cells. The bottom panels show the ablation of one third of the frontmost part of the group.

Movie S5. Trunk Neural Crest Migration in Zebrafish, Related to Figure 3 The first panel shows a maximal projection of TNC cells of the segments 8–9 in a time-lapse movie from a Sox10:mG embryo. This is followed by three panels of nuclear fluorescence projection overlaid by the tracking of leader cells (at the front of the chain), representative follower cells (trailing the leader), and representative premigratory cells (before somite invasion). The images were taken every 5 min, for a total duration of 650 min (lateral view, dorsal top, and anterior left).

Movie S6. Trunk and Cranial Neural Crest Cell-cell Contact in Zebrafish, Related to Figure 3 The movie shows a maximal projection of membrane bound GFP of a Sox10:mG embryo, showing the protrusions dynamics upon cell-cell contact between two cells. The left panel shows CNC cells from seven cells analyzed from two embryos, and the right panel shows TNC cells from eight cells analyzed from three embryos. The arrowheads point to retracting protrusions. The images were taken at a 30 s interval, for a total duration of 18 min (lateral view, dorsal top, and anterior left).

Movie S7. Leader and Follower Cell Division in Zebrafish, Related to Figure 4 The movie shows a maximal projection of a time-lapse movie from a Sox10:mG embryo, showing a dividing follower cell, top left, and dividing leader cell, bottom left panel. The right panels display their respective overlaid tracking. The blue track follows the dividing cell and the red and green tracks trail the resulting daughter cells. The cyan track shows the cell following the dividing cell. The images were taken at 5 min intervals (follower cell total time 80 min) (leader cell 140 min) (lateral view, dorsal top, and anterior left).

Movie S8. Trunk Neural Crest Cell Intermixing during Migration in Zebrafish, Related to Figure 4 The first panel shows a maximal projection of a time-lapse movie from segments 8–9 from a Sox10:mG embryo. The second panel shows an overlay of the nuclear fluorescence and the track of leader and follower cells together. The images were taken every 5 min, for a total duration of 495 min (lateral view, dorsal top, and anterior left).

Movie S9. Chain Migration Is Rescued by a Premigratory Cell when the Leader Is Abnormally Arrested at Cytokinesis in Zebrafish, Related to Figure 4 The first panel shows a maximal projection of a time-lapse movie from a Sox10:mG embryo. The second panel shows an overlay of the nuclear fluorescence and the track of the arrested leader, follower of the chain, and rescuing premigratory cells together. The images were taken every 5 min, for a total duration of 570 min (lateral view, dorsal top, and anterior left).

Movie S10. Trunk Neural Crest Norphology in Zebrafish, Related to Figure 5 The movie shows 3D models of leader, follower, and premigratory cells (lateral view, dorsal top, and anterior left, 360° rotation).

Movie S11. Trunk Neural Crest Leader Cell Ablation in Zebrafish, Related to Figure 6 The movie shows two examples of leader cell laser ablation experiments. The sequence for both examples: cartoon representation of the experiment, followed by the preablation and postablation snapshots and time-lapse movie. The segments 8–9 are shown. The left panel shows a maximal Z projection of fluorescent channels from a Sox10:mG embryo. The right panel shows a maximal Z projection of nuclear fluorescence overlaid by the tracks of leader and follower cells. The images were taken every 5 min (lateral view, dorsal top, and anterior left).

Movie S12. Trunk Neural Crest Ablation of Follower Cells: Gap and Follower Ablation in Zebrafish, Related to Figure 7 The movie shows two examples of follower cell laser ablation generating a gap and one example of follower cell laser ablation that maintains cell contact continuity in the chain. The sequence for every example: cartoon representation of the experiment, followed by the preablation and postablation snapshots, and time-lapse movies. The segments 8–9 are shown. The left panel shows a maximal Z projection of fluorescent channels from a Sox10:mG embryo. The right panel shows a maximal Z projection of nuclear fluorescence overlaid by the tracks of leader and follower cells. The images were taken every 5 min (lateral view, dorsal top, and anterior left).

Document S2. Article plus Supplemental Information
