# Supplementary material for: Leader Cells Define Directionality of Trunk, but Not Cranial, Neural Crest Cell Migration
Source: Cell Rep. 2016 May 19;15(9):2076–88. doi: 10.1016/j.celrep.2016.04.067 (PMC4893160; doi:10.1016/j.celrep.2016.04.067)
Supplement: Document S1. Supplemental Experimental Procedures and Figures S1–S3 [file mmc1.pdf]

Cell Reports, Volume *15*

Supplemental Information

Leader Cells Define Directionality of Trunk, but Not Cranial, Neural Crest Cell Migration

Jo Richardson, Anton Gauert, Luis Briones Montecinos, Lucía Fanlo, Zainalabdeen Mohammed Alhashem, Rodrigo Assar, Elisa Marti, Alexandre Kabla, Steffen Härtel, and Claudia Linker

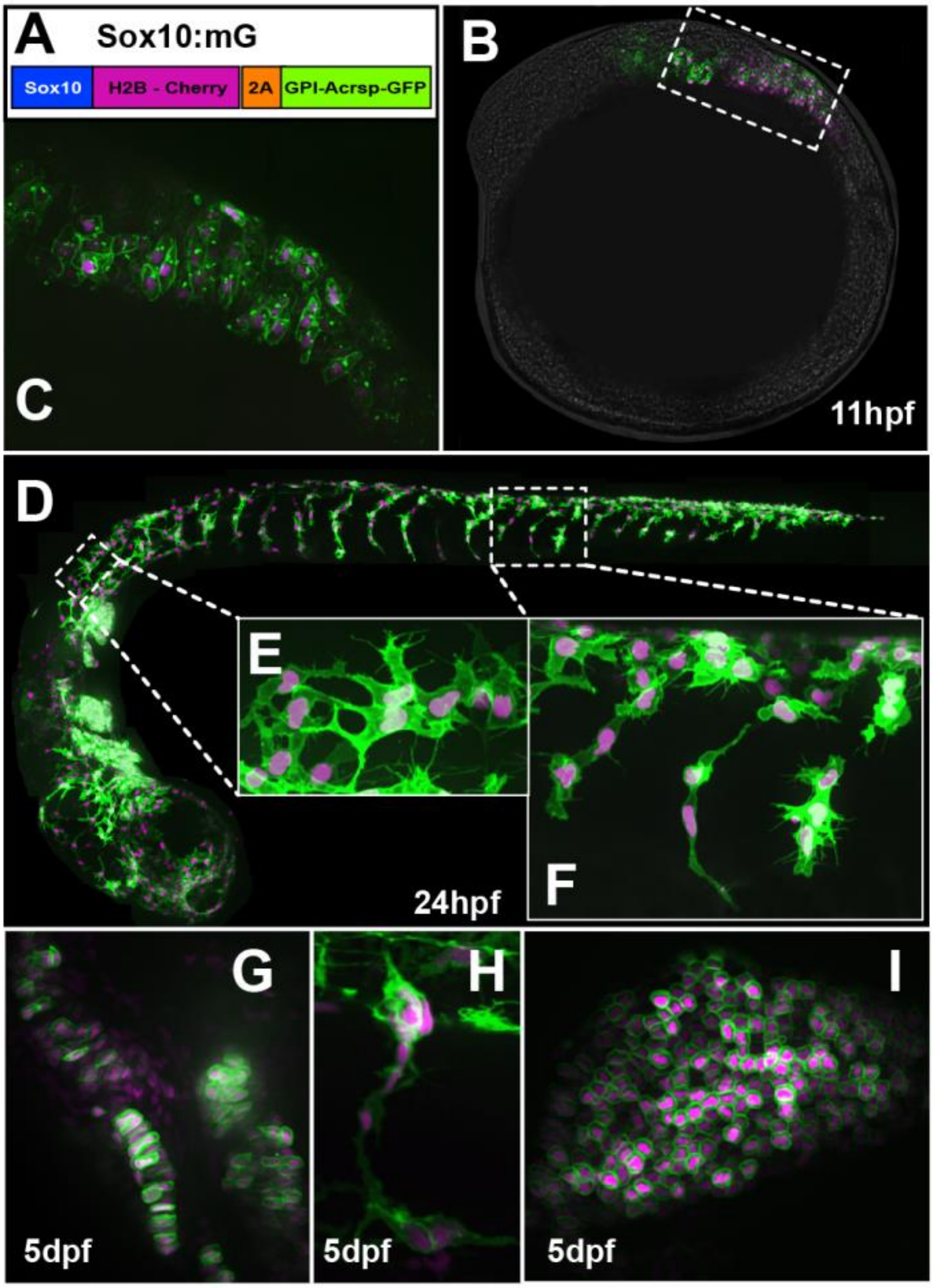

Figure S1, related to Figure 1. Neural crest reporter line Sox10:mG
(A) Schematic of the sequences used to generate the Sox10:mG line. (B-C) Fluorescently labelled NCCs visible at 11hpf in the anterior most part of the embryo, (D-F) at 24hpf following stereotypical migratory routes in the head and trunk. (G-I) Label is maintained in NCs derivatives at 5dpf, (G) cranial cartilage cells, (H) trunk dorsal root ganglia and associated glia and (I) pectoral fin. Anterior to the left, dorsal to the top. B-F and H lateral views. G coronal optical section through the lower jaw. I dorsal view.

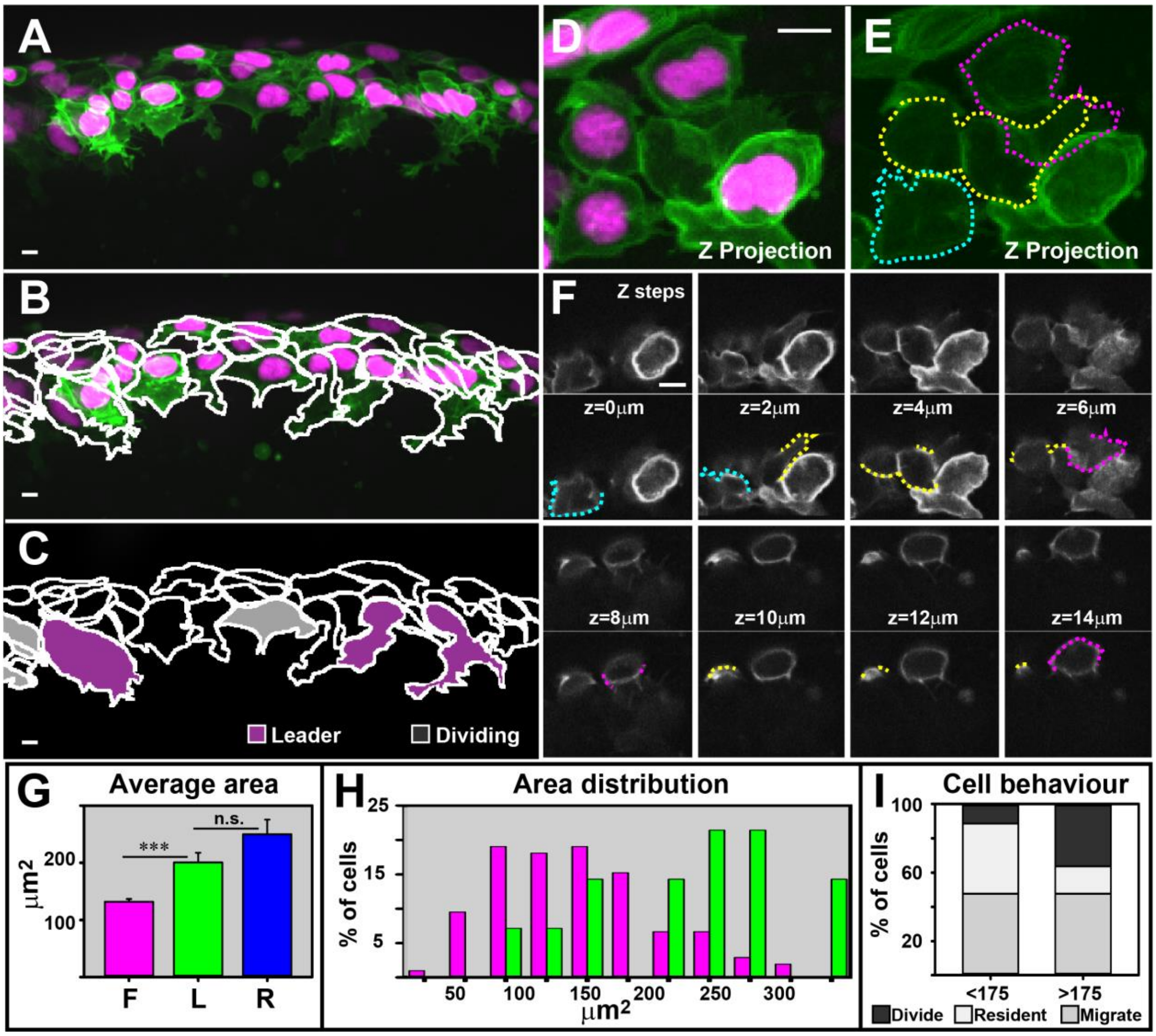

Figure S2, related to Figure5. Leader and follower cells present characteristic morphologies before the initiation of migration.

(A) Picture of representative embryo premigratory area before the initiation of migration, (B) with cell outlines and (C) with leaders in purple and cell that divide within 90' in grey (excluded from analysis). (D-F) Example of the manner in which cell outline was defined. Magnification bar 10μm. (D) Magnified picture of representative embryo premigratory area before the initiation of migration, (E) with cell outlines and (F) z-planes corresponding to the image. Top panel shows the membrane fluorescence image, bottom panel the outline of the membrane draw for each picture, that were all combined in E. Magnification bar 5μm. (G) Average areas of follower and leader cells are significantly different. Leader and rescuing cells area are not significantly different. (H) Area distribution of premigratory cells. (I) Behavior of small >175μm or big <175μm cells after leaders have initiated their migration. Half of the big or small cells migrate as followers. From the cells that remain premigratory big cells tend to divide more that small cells. n numbers F=105, L=14, 6 embryos. Error bars SEM. Lateral views, anterior left, dorsal top. All error bars 10. See also Movies S5.

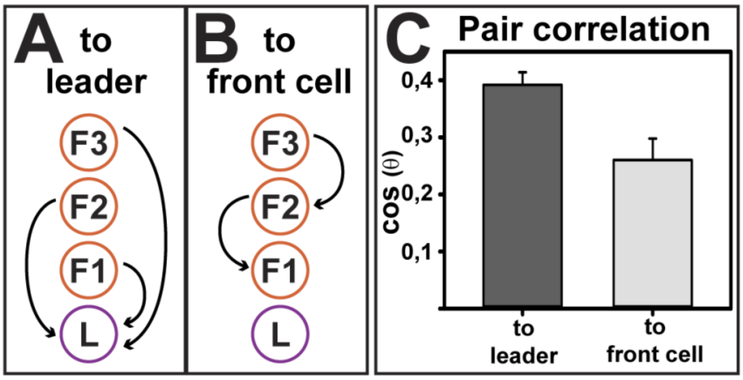

Figure S3, related to Figure 3. All cells follow the leader: pair correlation analysis

(A) Diagram of pair correlation to leader. Arrow indicates comparison of angle of migration between every F: follower and its L: leader. (B) Diagram of pair correlation to the front neighbor. Arrow indicates comparison of angle of migration between F: follower and the follower in front of it. (C) Average of the correlation coefficients are significantly different P=<0.001, error bars SEM. Leaders n=8, followers n=16, 4 embryos.

Supplemental movies legends

Movie S1, related to Figure 1. Cranial neural crest migration in chick embryos.

First panel, maximal projection of pre-otic CNCs time lapse movie from a chick embryo in which the neural tube has been electroporated with H2B-GFP plasmid. Followed by an overlay with the tracking of representative 1st, 2nd, 3rd and 4th quartile separated by the time of migration initiation. Arrows point to cells that migrate against the overall directionality. Last panel overlay of the tracking of the first cells that initiate migration, 1st quartile, and all other cells that finish their movement at the front of the group. Asterisks indicate cells that initiate migration at the front but are left behind over the course of the experiment. Images were taken every 3 min, for a total duration of 237 minutes. Dorsal view, anterior left.

Movie S2, related to Figure 2. Cranial neural crest migration in zebrafish embryos.

First panel, maximal projection of post-otic CNCs time lapse movie from a Sox10:mG embryo. Followed by three panels of nuclear fluorescence projection overlaid by tracking of representative front cells (presenting membrane to the leading edge), middle cells (surrounded by other NCs) and back cells (presenting membrane to the rear of the group). Images were taken every 5 min, for a total duration of 400 min. Lateral view, dorsal top, anterior left.

Movie S3, related to Figure 2. Cranial neural crest cell intermixing during migration in zebrafish.

First panel, maximal projection of post-otic CNCs time lapse movie from a Sox10:mG embryo. Second panel overlay of the nuclear fluorescence and the track of cells that initiate their migration at the front of the group and all other cells that finish their movement at the front of the group. Third panel overlay of the nuclear fluorescence and the track of cells that initiate their migration at the front of the group. Fourth panel overlay of the nuclear fluorescence and the track of cells that initiate their migration at the middle of the group and finish at the front. Fifth panel overlay of the nuclear fluorescence and the track of cells that initiate their migration at the back of the group and finish at the front.

Images were taken every 5 min, for a total duration of 495 min. Lateral view, dorsal top, anterior left.

Movie S4, related to Figure 2. Cranial neural crest leader cell ablation in zebrafish.

Top panels right maximal projection of the fluorescent channels. Left panels, nuclear fluorescence overlaid with the track of cells that were at the front of the group after the ablation procedure or that finished their migration at the front of the group. First frame pre-ablation picture; blue indicates the nuclei that will be ablated. Second frame the initiation of the movie, blue dashed line indicates the membrane outline before the ablation procedure. Top panels ablation of the first row of front cells. Bottom panels ablation of one third of the frontmost part of the group.

Movie S5, related to Figure 3. Trunk neural crest migration in zebrafish.

First panel, maximal projection of TNCs of segments 8-9 time lapse movie from a Sox10:mG embryo. Followed by three panels of nuclear fluorescence projection overlaid by tracking of leader cells (at the front of the chain), representative follower cells (trailing the leader) and representative premigratory cells (before somite invasion). Images were taken every 5 min, for a total duration of 650 min. Lateral view, dorsal top, anterior left.

Movie S6, related to Figure 3. Trunk and Cranial neural crest cell-cell contact in zebrafish.

Maximal projection of membrane bound green fluorescent protein of a Sox10:mG embryo, showing the protrusions dynamics upon cell-cell contact between two cells. Left panel CNCs 7 cells analyzed from 2 embryos; right panel TNCs 8 cells analyzed from 3 embryos. Arrowheads point to retracting protrusions. Images were taken at a 30 seconds interval, for a total duration of 18 min. Lateral view, dorsal top, anterior left.

Movie S7, related to Figure 4. Leader and follower cell division in zebrafish.

Maximal projection of a time lapse movie from a Sox10:mG embryo, showing a dividing follower cell, top left, and dividing leader cell, bottom left panel. Right

panels display their respective overlaid tracking. Blue track follows the dividing cell, red and green tracks trail the resulting daughter cells. Cyan track shows the cell following the dividing cell. Images taken at 5 min interval. Follower cell total time 80 min. Leader cell 140 min. Lateral view, dorsal top, anterior left.

Movie S8, related to Figure 4. Trunk neural crest cell intermixing during migration in zebrafish.
First panel, maximal projection of time lapse movie of segments 8-9 time lapse movie from a Sox10:mG embryo. Second panel overlay of the nuclear fluorescence and the track of leader and follower cells together. Images were taken every 5 min, for a total duration of 495 min. Lateral view, dorsal top, anterior left.

Movie S9, related to Figure 4. Chain migration is rescued by a premigratory cell when leader is abnormally arrested at cytokinesis in zebrafish.
First panel, maximal projection of time lapse movie from a Sox10:mG embryo. Second panel overlay of the nuclear fluorescence and the track of the arrested leader, follower of the chain and rescuing premigratory cells together. Images were taken every 5 min, for a total duration of 570 min. Lateral view, dorsal top, anterior left.

Movie S10, related to Figure 5. Trunk neural crest morphology in zebrafish.
Three dimensional models of leader, follower and premigratory cells. Lateral view, dorsal top, anterior left, 360° rotation.

Movie S11, related to Figure 6. Trunk neural crest leader cell ablation in zebrafish.
Two examples of leader cell laser ablation experiments. Sequence for both examples: cartoon representation of the experiment, followed by the pre-ablation and post-ablation snapshots and time lapse movie. Segments 8-9 shown. Left panel, maximal Z projection of fluorescent channels from a Sox10:mG embryo. Right panel maximal Z projection of nuclear fluorescence

overlaid by the tracks of leader and follower cells. Images were taken every 5 min. Lateral view, dorsal top, anterior left.

Movie S12, related to Figure 7. Trunk neural crest ablation of follower cells: gap and follower ablation in zebrafish.

Two examples of follower cell laser ablation generating a gap and one example of follower cell laser ablation that maintains cell contact continuity in the chain.

Sequence for every example: cartoon representation of the experiment, followed by the pre-ablation and post-ablation snapshots, and - time lapse movies. Segments 8-9 shown. Left panel, maximal Z projection of fluorescent channels from a Sox10:mG embryo. Right panel maximal Z projection of nuclear fluorescence overlaid by the tracks of leader and follower cells. Images were taken every 5 min. Lateral view, dorsal top, anterior left.

Supplemental Materials and Methods:

Chick embryo *ex ovo* electroporation and EC culture

Fertilized eggs from White-Leghorn chickens were incubated at 38.5ºC in an atmosphere of 70% humidity. Embryos were staged according to Hamburger and Hamilton (1992) and electroporated with Histone 2B-GFP Clontech purified pCS2 plasmid DNA diluted in H2O with 50ng/ml Fast Green (Sigma) at a concentration of 0.5-1µg/µl. Plasmid DNA was injected into the lumen of HH8-9 neural tubes (4 to 6 somites) under a Leica MZ6 scope. Electrodes were placed either side of the future hindbrain and electroporation carried out ex ovo using an Intracel Dual Pulse TSS-100 electroporator delivering five to seven 50 ms square pulses of 20V.Embryos were electroporated with Histone-2B RFP and membrane-GFP in pCS2 to measure the average cell diameter.

After electroporation EC culture were performed (Chapman et al., 2001; Streit, 2008). 225ul agar-albumin mix was added to Ibdi uncoated µ-Dish (Ibdi uncoated µ-Dish 35mm, high, cat No 81151) and allowed to solidify at room temperature for 30 to 40 minutes. Embryos were attached to Whatman filter paper circles (Cat No 10.312.611) and mounted ventral-side up. A wipe moistened in Pannett-Compton buffer was placed inside the µ-Dish to keep humidity. Embryos were culture for 8 hours before imaging. Imaging was performed dorsally every 3' for 10-12hours using a ZEISS LSM780 system, 100µm z-stacks with 2.5µm z-steps were taken.

Generation of the Sox10mG Transgenic Line

The 4.9kb *Sox10* promoter (Carney et al., 2006) drives expression of multicistronic open reading frame for H2B-monomeric Cherry (chromatin-label) and membrane tagged GFP (GPI), separated by the 2A viral peptide

(Shioi et al., 2011). Cloning and trangenesis was performed according to the Tol2kit protocols.

Chick Time-lapse imaging

Embryos were imaged using a ZEISS LSM780 microscope at 37ºC, a 10X (Plan-Apochromat NA 0.45 air, working distance 2mm) or a 25X (Plan-Apochromat NA 0.75 water immersion, working distance 0.55 mm) objectives, and laser lines 488nm. 100µm z-stacks with 2.5µm z-steps were taken every 3 minutes for 10-12hours.

Zebrafish Time-lapse imaging

Embryos were anesthetized with 3-amino benzoic acid ethyl ester (ethyl 3-aminobenzoate; 0.0168% Tricaine) and immobilized in 1.2% low melting point agarose. Lateral views of somite 7-9 at 16 hpf, or 18-22 hpf for ablation experiments, were imaged using a PerkinElmer ULTRAVIEW Vox system and a 40x long working distance lens (1 NA), the 488 and 563nm laser lines. Embryos were imaged in E3 media buffered with 5 mM HEPES (pH 7.2) and anaesthetic. 70µm z-stacks with 2µm z-steps were taken every 5 minutes for 16-18hours. Membrane dynamics and 3D models a 1µm z-step was used, imaging every 30 seconds.

Laser Ablations

Laser ablations were performed using a MicroPoint (Andor) nitrogen laser system with 440 nm Coumarin dye, which was aimed to the nuclei of the targeted cell identified by RFP expression. Laser power was set to maximum without any grey filters attenuation. Four pulses of 80Hz were applied to two different locations of

the same nuclei. In our hands, the laser power was optimal when single laser pulses caused slight movement and, occasionally, a small air bubble. The integrity of the cells surrounding the targeted cell was monitored by imaging all membranes in BODIPY®TR methyl ester labelled embryos (Cooper et al., 2005). In brief, embryos were incubated for 1h in 100μM BODIPY®TR methyl ester with 2%DMSO solution at 28.5°C, washed tree times with HEPES-buffered E3 media before ablations were performed.

Tracking of cell nuclei and computational analysis of migration pattern

All images were corrected for drift; this was accomplished by calculating necessary correction for the bright field channel drift, the same correction was then applied to the fluorescent channels.

Drift correction: A single Z plain bright field image that maintained somite borders in focus during the entire length of the movie was selected. This sequence was corrected for using the ImageJ Register Virtual Stack Slices plugin (http://imagej.net/Register_Virtual_Stack_Slices). The correction matrix generated for each time point was then applied to every Z planes at that time point for the fluorescent channels.

Tracking: Corrected images of cell nuclei were then tracked semi-automatically in 3D using View5D plugin (Rainer Heintzmann, King's College, London) of ImageJ (Schneider et al., 2012). This produced a txt file with all cell coordinates in X,Y and Z.

Cell separator: The coordinates of individual cells were separated using the Cell separator Matlab script (see supplementary data files).

Directionality ratio, linear trajectory and path distance: were calculated using excel macros as in Gorelik and Gautreau, 2014.

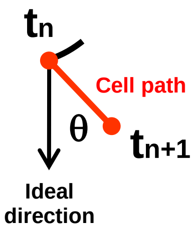

Directionality correlation: Compares the direction of a cell path to the ideal direction of migration. The cosine of the angle between a cell's path and the ideal direction of migration (represented by a straight vertical line) was calculated for each time step and average

Pair Correlation: Compares the direction of two cell trajectories. The cosine of the angle between two cells' path (A and B) was calculated for each time step and average over all time points. Cells coordinates were first separated using the cell separator (above) then pair correlation was calculated using the pair correlation Matlab script (see supplementary data files). The time windows at which one cell is the front neighbor of another was visually decided according to the image series. The pair correlation data was collated accordingly.

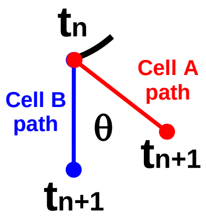

Centre of mass tracking

Cells were manually outlined from 2D projections of time-lapse images and transformed into binary images using ImageJ. Track of the center of mass was obtained using the MTrack2 plugin. A running average of three time points was plotted.

Generation of 3D cell models

The segmentation process and generation of 3D models were performed using SCIAN-Lab image processing software based on IDL 7.1.2 (Interactive Data Language, Exelisvis, CO, USA). Cells were segmented in each image to create 2D binary masks (pixels) that were connected in the z-axis for the generation of 3D cell models (voxels). Cell volume was approximated by voxel counting,

elongation index was calculated on the basis of the first principal axis (A1) and the second principal axis (A2) following (Russ, 2006) as 1-A2/A1. For 3D cell visualization, we applied an active contour model described in Härtel et al., 2007.

2D and 3D Tracking visualization

The Manual tracking plugin from ImageJ (FIJI) was use for visualization of 2D cell trajectories. Nuclei movement coordinates obtained from View5D were converted into Manual tracking plugin format using the Manual tracking convertor Matlab script (see supplementary data files).

IMARIS software (Bitplane) was used for visualization of the 3D cell trajectories: (i) coordinates of each track were transform to binary images using the Trajectories Matlab script (see supplementary data files), (ii) each coordinate was converted into a pixel with full intensity while pixels corresponding to background were set to zero, (iii) a new 3D binary stack was generated and opened with IMARIS to perform visualization of the 3D trajectories using spots tool at the automatically detected threshold level.

Protrusion quantification

2D projections of time-lapse images were produced for each cell. All cells were aligned according to their direction of migration. Straight lines were manually drawn from the center of the nuclei to the distal most point of every protrusion in the cell. Angles and number of protrusions were measure using ImageJ.

Area calculation

Areas were calculated from manually outlined cells in 2D projections using

ImageJ software.

Extension retraction analysis

For the extension-retraction analysis, cells were manually outlined from 2D projections of time-lapse images and transformed into binary images using ImageJ. Frames were either subtracted from the previous frame (to highlight retraction, red) or from the subsequent frame (to highlight extension, blue).

Angle division calculation

The plane of division was manually drawn over 2D projection image and its angle was measured using ImageJ.

MSD calculation

The MSD function can be used to classify migratory patterns (Gorelik and Gautreau, 2014). Ballistic motion (cells going straight) is characterised by MDS(Dt) = v^2 Dt^2. Stochastic motion (random, uncorrelated displacements from one time point to the next) is characterised by MSD(Dt) = D Dt.

MSD(Dt) = < (r(t+Dt) - r(t)) > (<x> = average of x)

MSD is a function of a time interval Dt.; r is the cell position vector at time t.

This is averaged over cells and time. Cell divisions interrupted trajectories.

Calculation of remnant movement

We first calculate the group velocity at each time point, integrate it over time to obtain the mean group trajectory (trend), and then subtract this trend from all individual trajectories obtaining the remnant movement. The group velocity at

any time t is determined by calculating the mean displacements of all the cells identified and tracked during the time interval (t-dt;t+dt), dt=5 mins.

Supplemental File sets:

Cell Separator, related to Figures 1, 2, 3, 4, 6 and 7. Matlab script that generates separated files for each tracked cell from View5D ImageJ plugin.

Manual Tracking Convertor, related to Figures 1, 2, 3, 4, 6 and 7. Matlab script that converts View5D tracking files into excel files readable by the Manual tracking plugin.

Pair Correlator, related to Figures S3. Matlab script that ccompares the direction of two or more cell trajectories.

Trajectories, related to Figures 1, 2, 3, 4, 6 and 7. Matlab script used to transform the coordinates of each track into binary images for visualization of View5D tracking in 3D using Imaris.

Supplemental References

Chapman, S.C., Collignon, J., Schoenwolf, G.C., Lumsden, A., 2001. Improved method for chick whole-embryo culture using a filter paper carrier. Dev. Dyn. Off. Publ. Am. Assoc. Anat. 220, 284–289. doi:10.1002/1097-0177(20010301)220:3<284::AID-DVDY1102>3.0.CO;2-5

Cooper, M.S., Szeto, D.P., Sommers-Herivel, G., Topczewski, J., Solnica-Krezel, L., Kang, H.-C., Johnson, I., Kimelman, D., 2005. Visualizing morphogenesis in transgenic zebrafish embryos using BODIPY TR methyl ester dye as a vital counterstain for GFP. Dev. Dyn. 232, 359–368. doi:10.1002/dvdy.20252

Gorelik, R., Gautreau, A., 2014. Quantitative and unbiased analysis of directional persistence in cell migration. Nat. Protoc. 9, 1931–1943. doi:10.1038/nprot.2014.131

Hamburger, V., Hamilton, H.L., 1992. A series of normal stages in the development of the chick embryo. 1951. Dev. Dyn. Off. Publ. Am. Assoc. Anat. 195, 231–272. doi:10.1002/aja.1001950404

Härtel, S., Jara, J., Lemus, C., Concha, M., 2007. 3D Morpho-Topological Analysis of Asymmetric Neuronal Morphogenesis in Developing Zebrafish. Comput. Model. Objects Represent. Images Fundam. Methods Appl. 215–220.

Russ, J.C., 2006. The Image Processing Handbook, Fifth Edition (Image Processing Handbook). CRC Press, Inc., Boca Raton, FL, USA.

Schneider, C.A., Rasband, W.S., Eliceiri, K.W., 2012. NIH Image to ImageJ: 25 years of image analysis. Nat. Methods 9, 671–675. doi:10.1038/nmeth.2089

Streit, A., 2008. EC culture: a method to culture early chick embryos. Methods Mol. Biol. Clifton NJ 461, 255–264. doi:10.1007/978-1-60327-483-8_17
