# Supplementary figures and images for: Leader Cells Define Directionality of Trunk, but Not Cranial, Neural Crest Cell Migration
Source: Cell Rep. 2016 May 19;15(9):2076–88. doi: 10.1016/j.celrep.2016.04.067 (PMC4893160; doi:10.1016/j.celrep.2016.04.067)

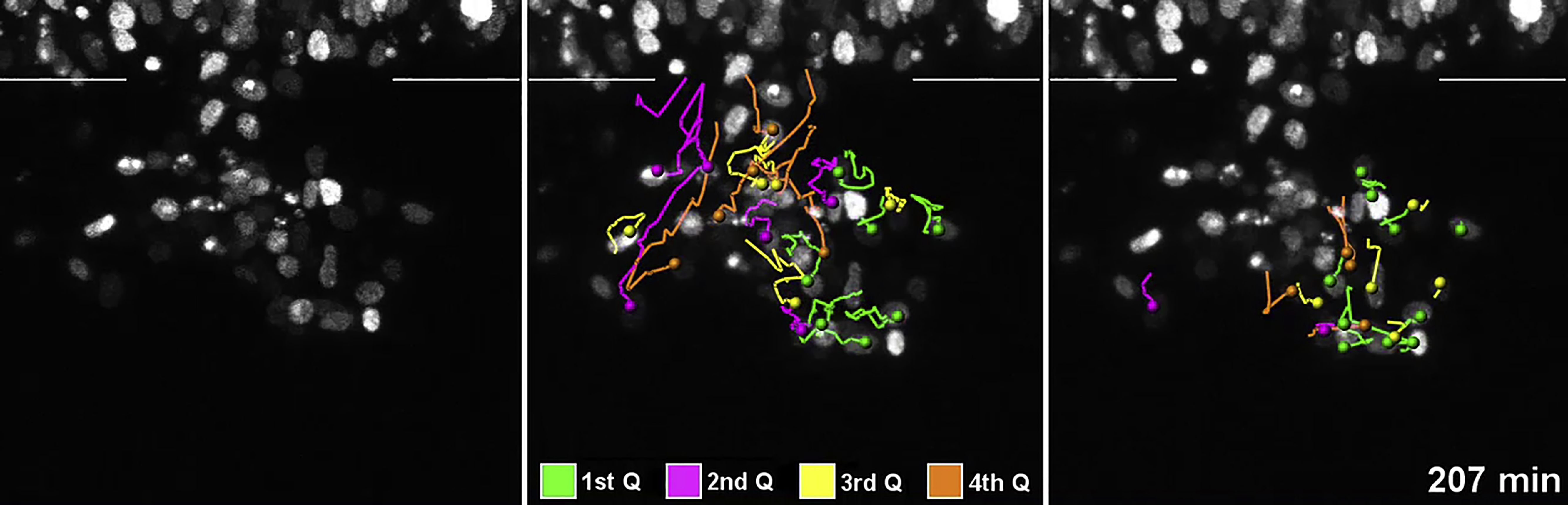

Supplement: Movie S1. Cranial Neural Crest Migration in Chick Embryos, Related to Figure 1 — The first panel shows a maximal projection of a preotic CNC cell time-lapse movie from a chick embryo in which the neural tube has been electroporated with H2B-GFP plasmid. This is followed by an overlay with the tracking of representative first, second, third, and fourth quartile separated by the time of migration initiation. The arrows point to cells that migrate against the overall directionality. The last panel shows the overlay of the tracking of the first cells that initiate migration, first quartile, and all other cells that finish their movement at the front of the group. The asterisks indicate cells that initiate migration at the front, but are left behind over the course of the experiment. The images were taken every 3 min, for a total duration of 237 minutes (dorsal view and anterior left). [file mmc2.jpg]

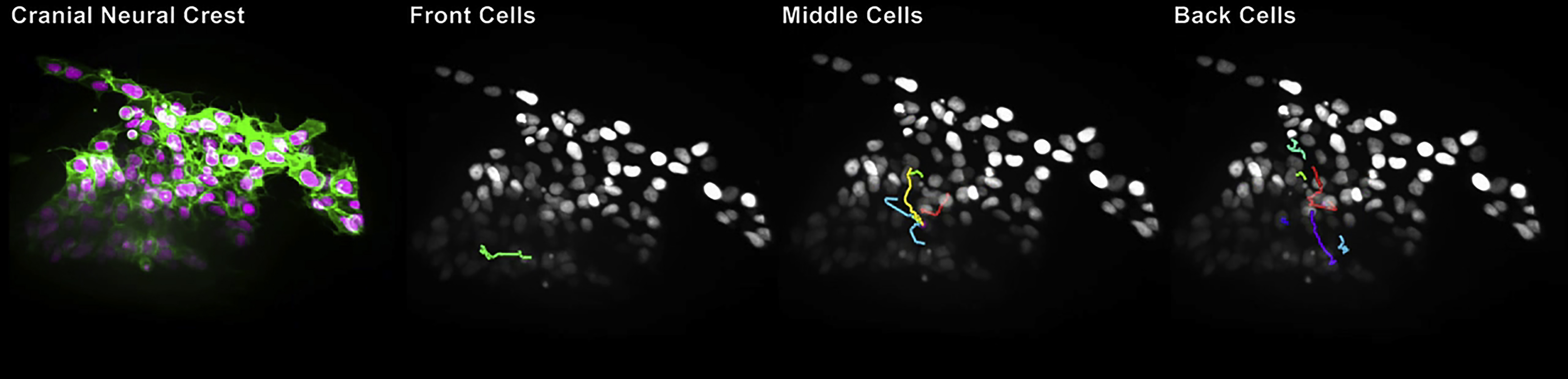

Supplement: Movie S2. Cranial Neural Crest Migration in Zebrafish Embryos, Related to Figure 2 — The first panel shows a maximal projection of a postotic CNC cell time-lapse movie from a Sox10:mG embryo. This is followed by three panels of nuclear fluorescence projection overlaid by the tracking of representative front cells (presenting membrane to the leading edge), middle cells (surrounded by other NC cells), and back cells (presenting membrane to the rear of the group). The images were taken every 5 min, for a total duration of 400 min (lateral view, dorsal top, and anterior left). [file mmc3.jpg]

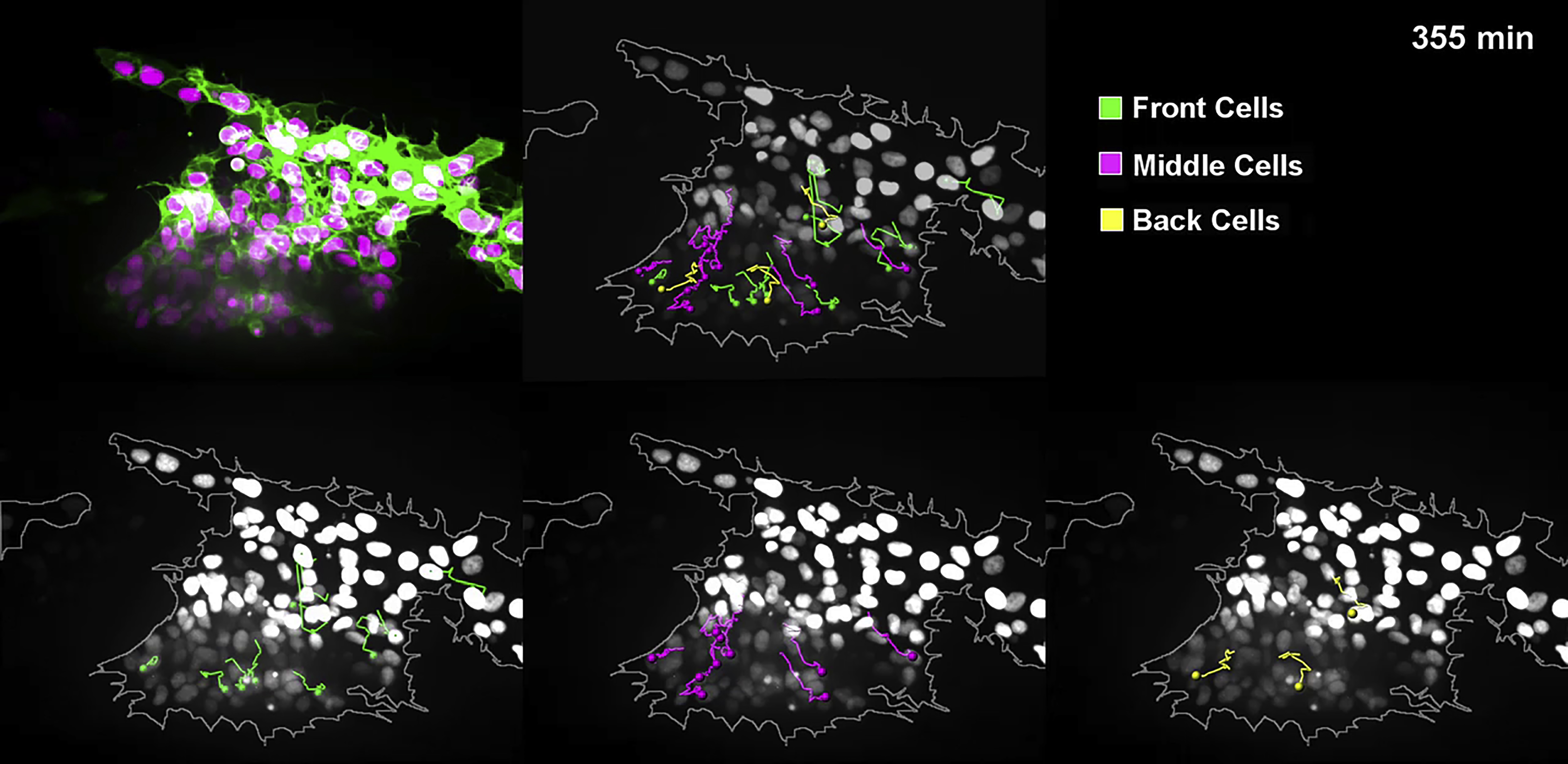

Supplement: Movie S3. Cranial Neural Crest Cell Intermixing during Migration in Zebrafish, Related to Figure 2 — The first panel shows a maximal projection of a postotic CNC cell time-lapse movie from a Sox10:mG embryo. The second panel shows an overlay of the nuclear fluorescence and the track of cells that initiate their migration at the front of the group and all other cells that finish their movement at the front of the group. The third panel shows an overlay of the nuclear fluorescence and the track of cells that initiate their migration at the front of the group. The fourth panel shows an overlay of the nuclear fluorescence and the track of cells that initiate their migration at the middle of the group and finish at the front. The fifth panel shows an overlay of the nuclear fluorescence and the track of cells that initiate their migration at the back of the group and finish at the front. The images were taken every 5 min, for a total duration of 495 min (lateral view, dorsal top, and anterior left). [file mmc4.jpg]

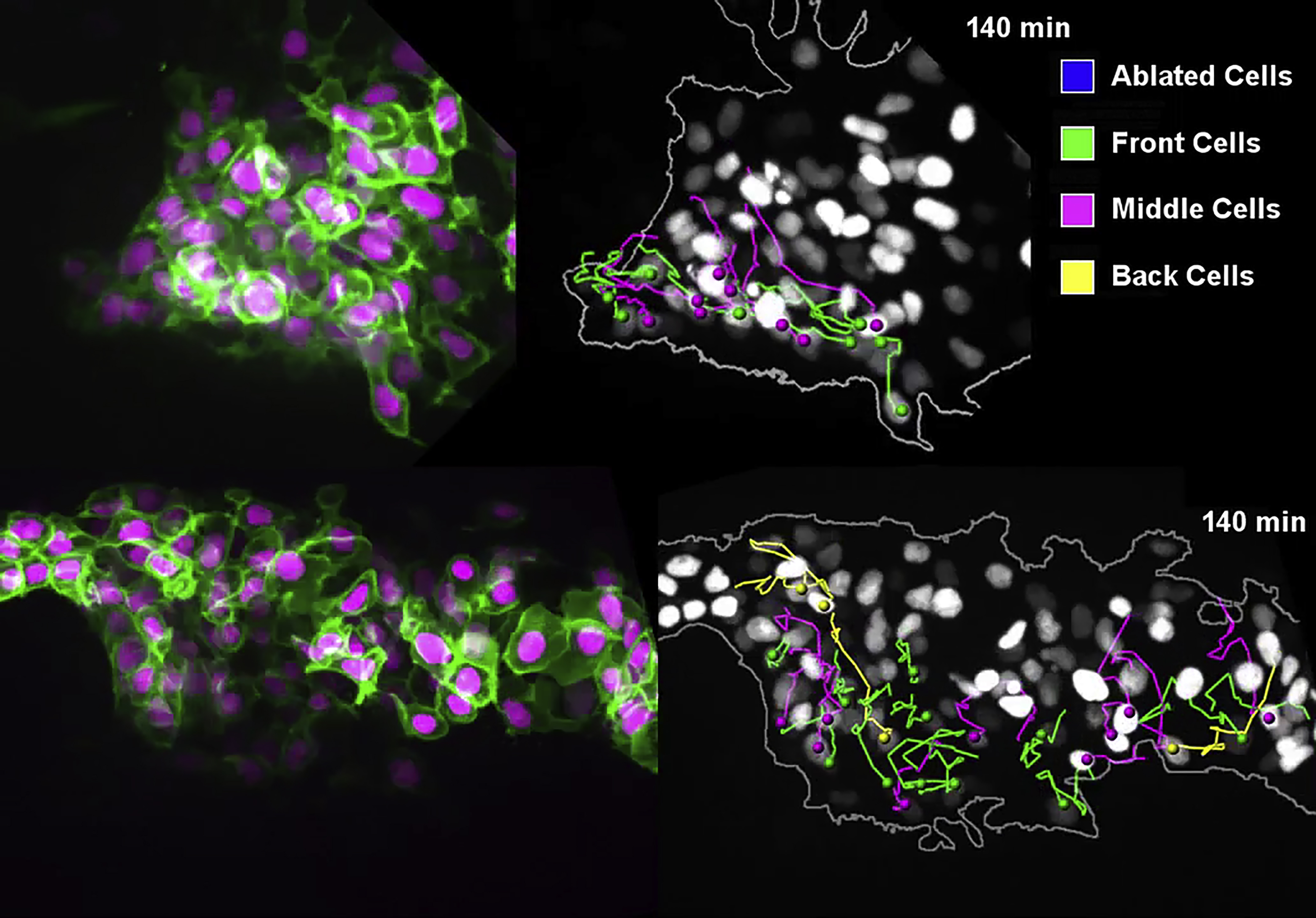

Supplement: Movie S4. Cranial Neural Crest Leader Cell Ablation in Zebrafish, Related to Figure 2 — The top panels on the right show a maximal projection of the fluorescent channels. The left panels show a nuclear fluorescence overlaid with the track of cells that were at the front of the group after the ablation procedure or that finished their migration at the front of the group. The first frame shows a preablation picture, and the blue indicates the nuclei that will be ablated. The second frame shows the initiation of the movie, and the blue dashed line indicates the membrane outline before the ablation procedure. The top panels show the ablation of the first row of front cells. The bottom panels show the ablation of one third of the frontmost part of the group. [file mmc5.jpg]

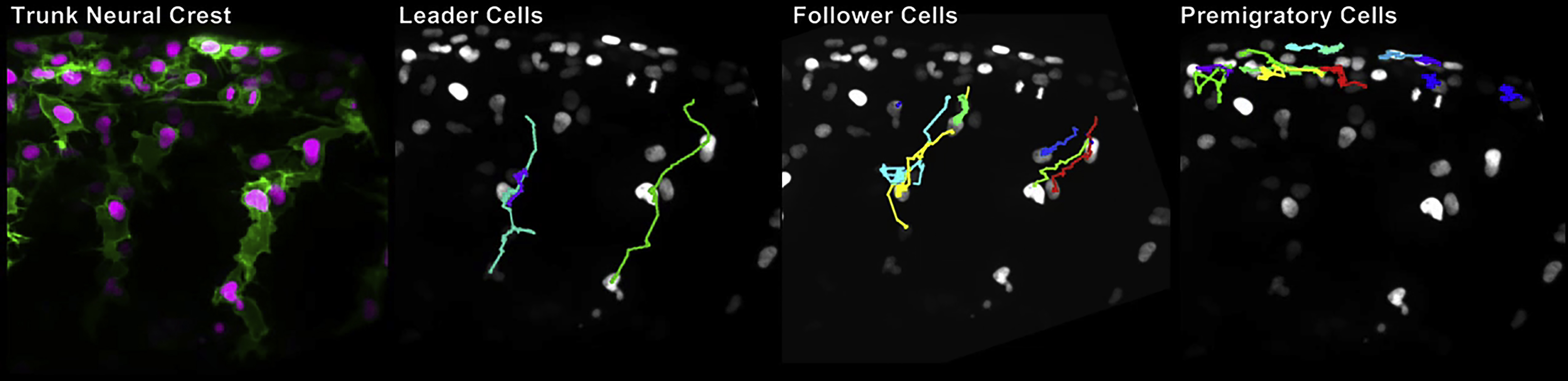

Supplement: Movie S5. Trunk Neural Crest Migration in Zebrafish, Related to Figure 3 — The first panel shows a maximal projection of TNC cells of the segments 8–9 in a time-lapse movie from a Sox10:mG embryo. This is followed by three panels of nuclear fluorescence projection overlaid by the tracking of leader cells (at the front of the chain), representative follower cells (trailing the leader), and representative premigratory cells (before somite invasion). The images were taken every 5 min, for a total duration of 650 min (lateral view, dorsal top, and anterior left). [file mmc6.jpg]

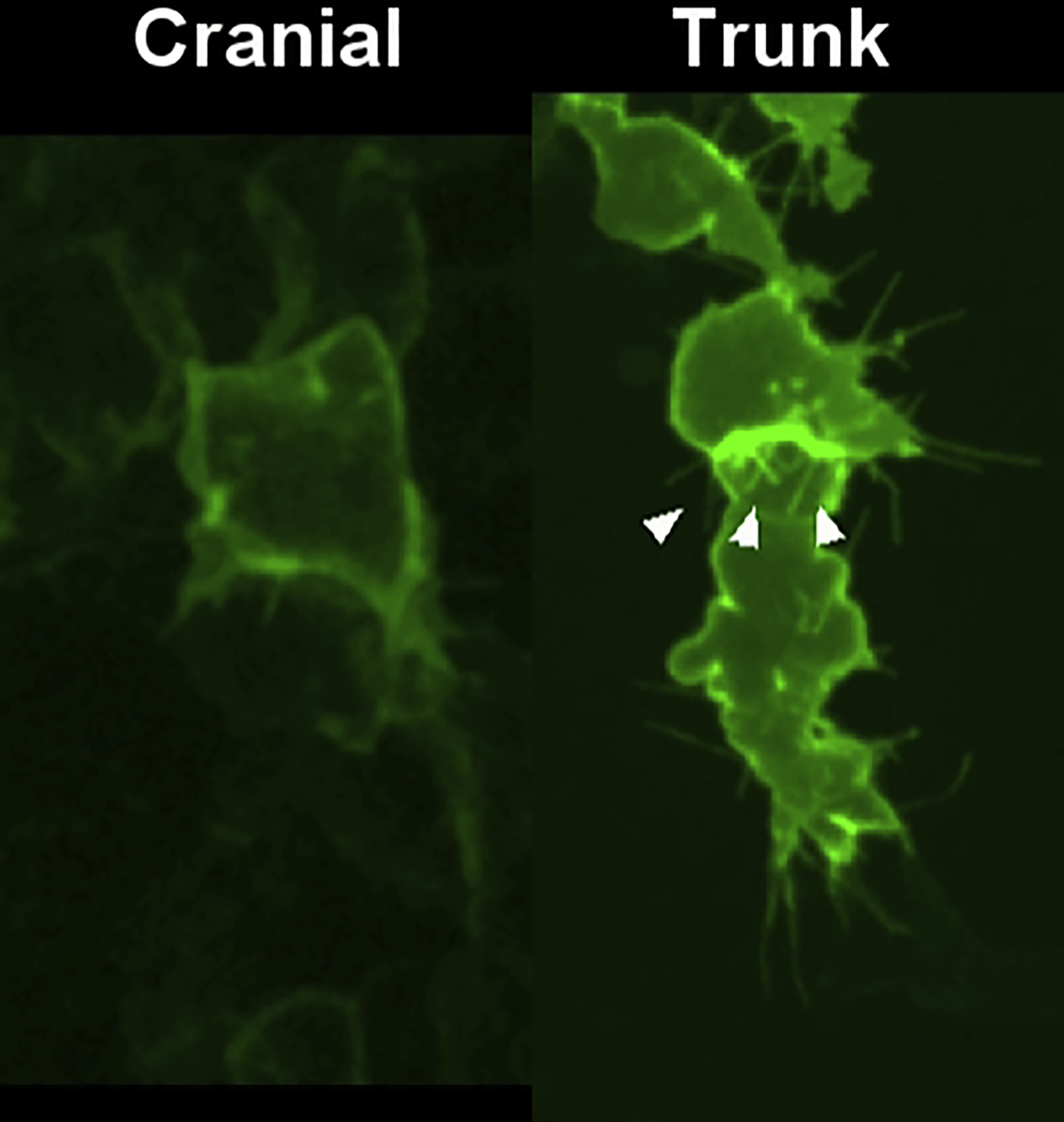

Supplement: Movie S6. Trunk and Cranial Neural Crest Cell-cell Contact in Zebrafish, Related to Figure 3 — The movie shows a maximal projection of membrane bound GFP of a Sox10:mG embryo, showing the protrusions dynamics upon cell-cell contact between two cells. The left panel shows CNC cells from seven cells analyzed from two embryos, and the right panel shows TNC cells from eight cells analyzed from three embryos. The arrowheads point to retracting protrusions. The images were taken at a 30 s interval, for a total duration of 18 min (lateral view, dorsal top, and anterior left). [file mmc7.jpg]

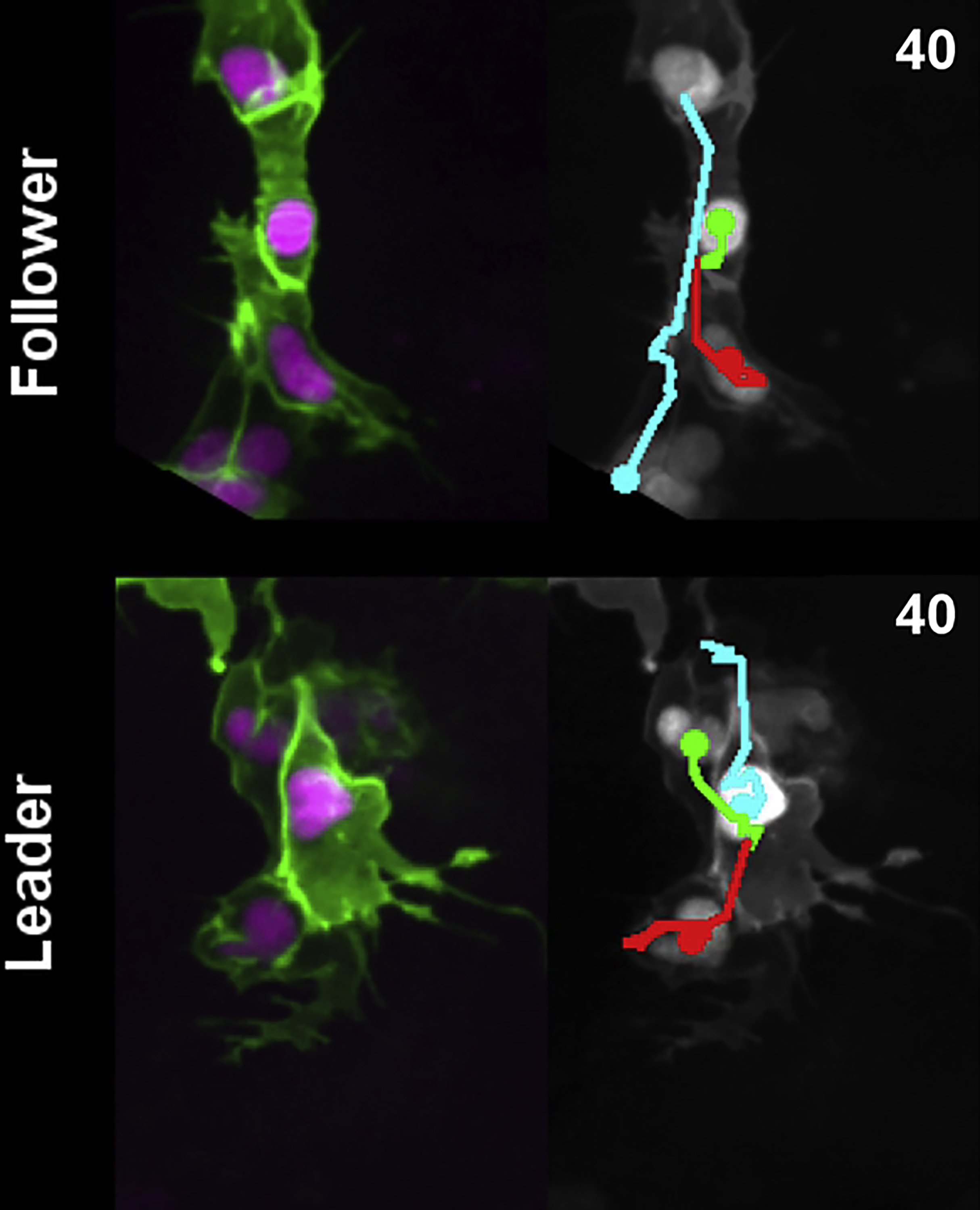

Supplement: Movie S7. Leader and Follower Cell Division in Zebrafish, Related to Figure 4 — The movie shows a maximal projection of a time-lapse movie from a Sox10:mG embryo, showing a dividing follower cell, top left, and dividing leader cell, bottom left panel. The right panels display their respective overlaid tracking. The blue track follows the dividing cell and the red and green tracks trail the resulting daughter cells. The cyan track shows the cell following the dividing cell. The images were taken at 5 min intervals (follower cell total time 80 min) (leader cell 140 min) (lateral view, dorsal top, and anterior left). [file mmc8.jpg]

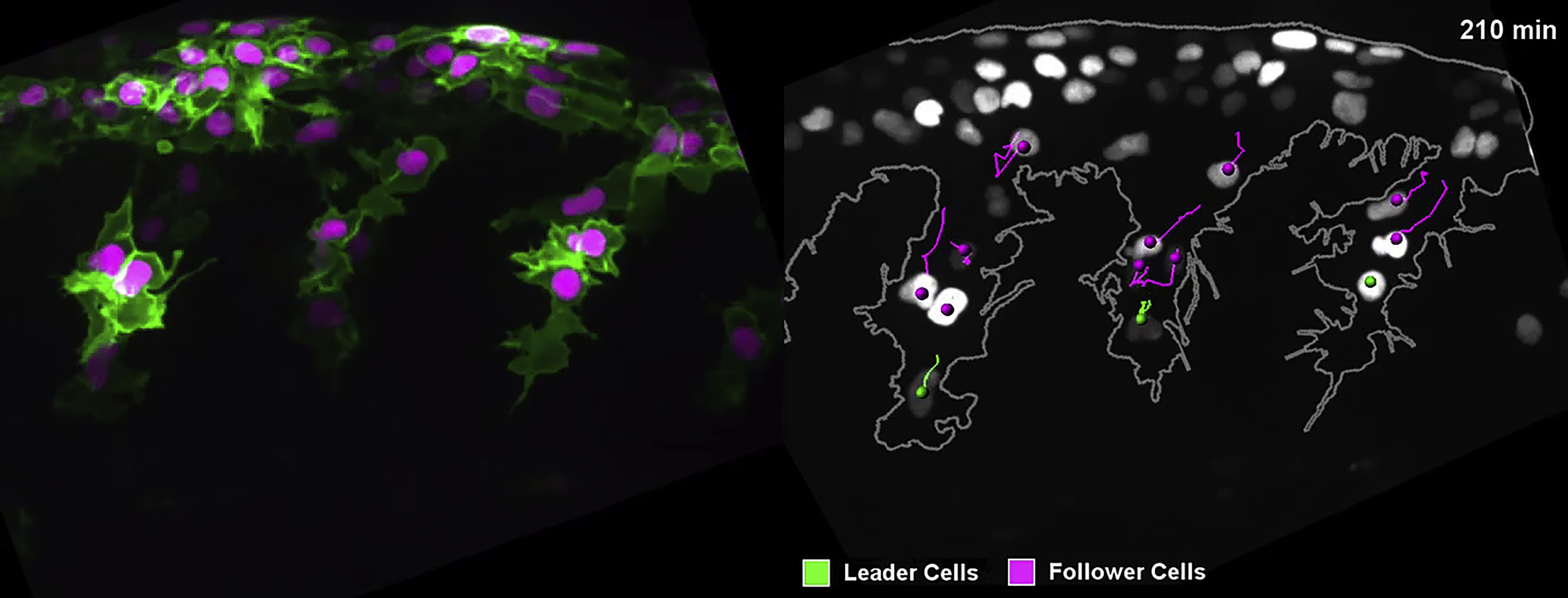

Supplement: Movie S8. Trunk Neural Crest Cell Intermixing during Migration in Zebrafish, Related to Figure 4 — The first panel shows a maximal projection of a time-lapse movie from segments 8–9 from a Sox10:mG embryo. The second panel shows an overlay of the nuclear fluorescence and the track of leader and follower cells together. The images were taken every 5 min, for a total duration of 495 min (lateral view, dorsal top, and anterior left). [file mmc9.jpg]

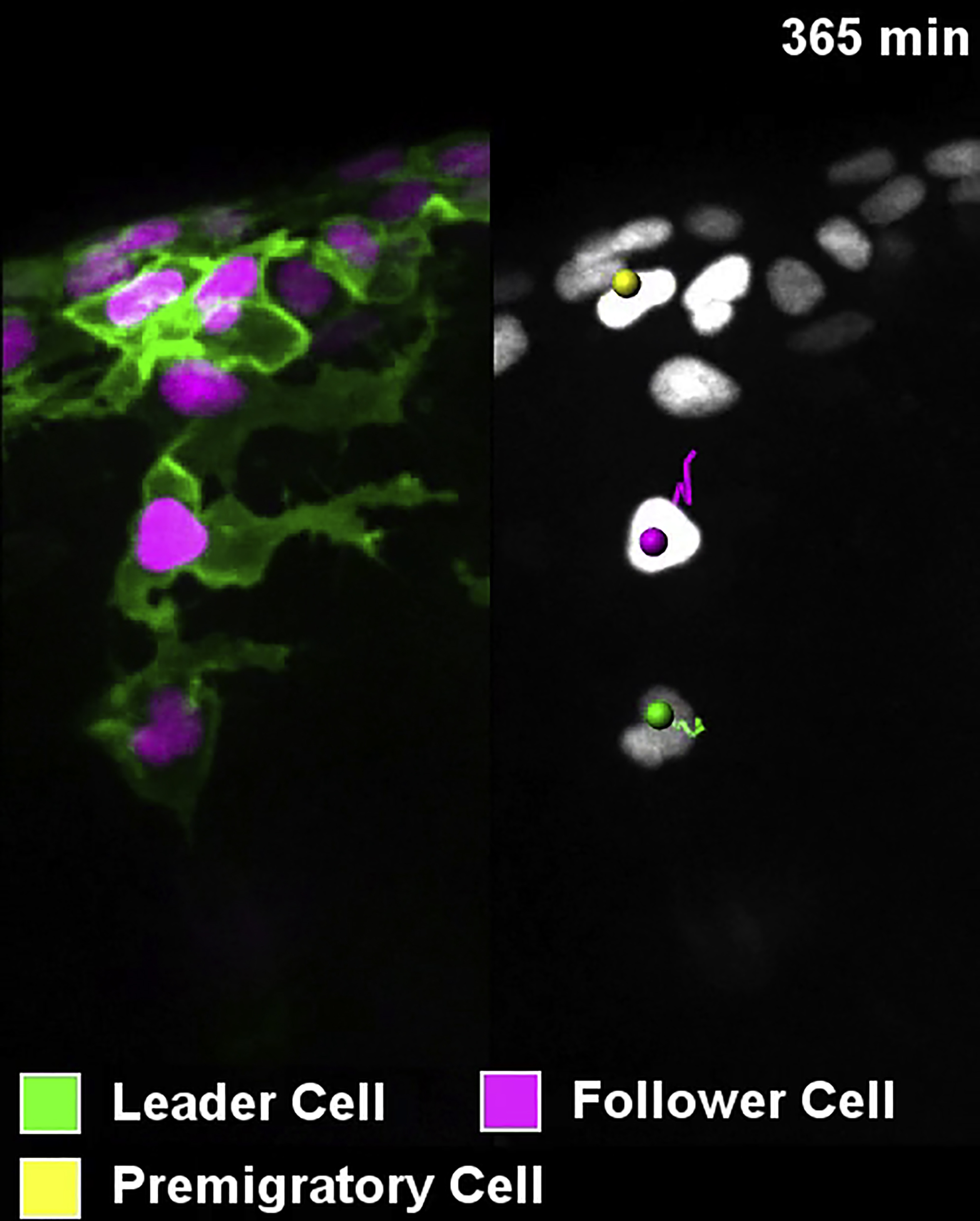

Supplement: Movie S9. Chain Migration Is Rescued by a Premigratory Cell when the Leader Is Abnormally Arrested at Cytokinesis in Zebrafish, Related to Figure 4 — The first panel shows a maximal projection of a time-lapse movie from a Sox10:mG embryo. The second panel shows an overlay of the nuclear fluorescence and the track of the arrested leader, follower of the chain, and rescuing premigratory cells together. The images were taken every 5 min, for a total duration of 570 min (lateral view, dorsal top, and anterior left). [file mmc10.jpg]

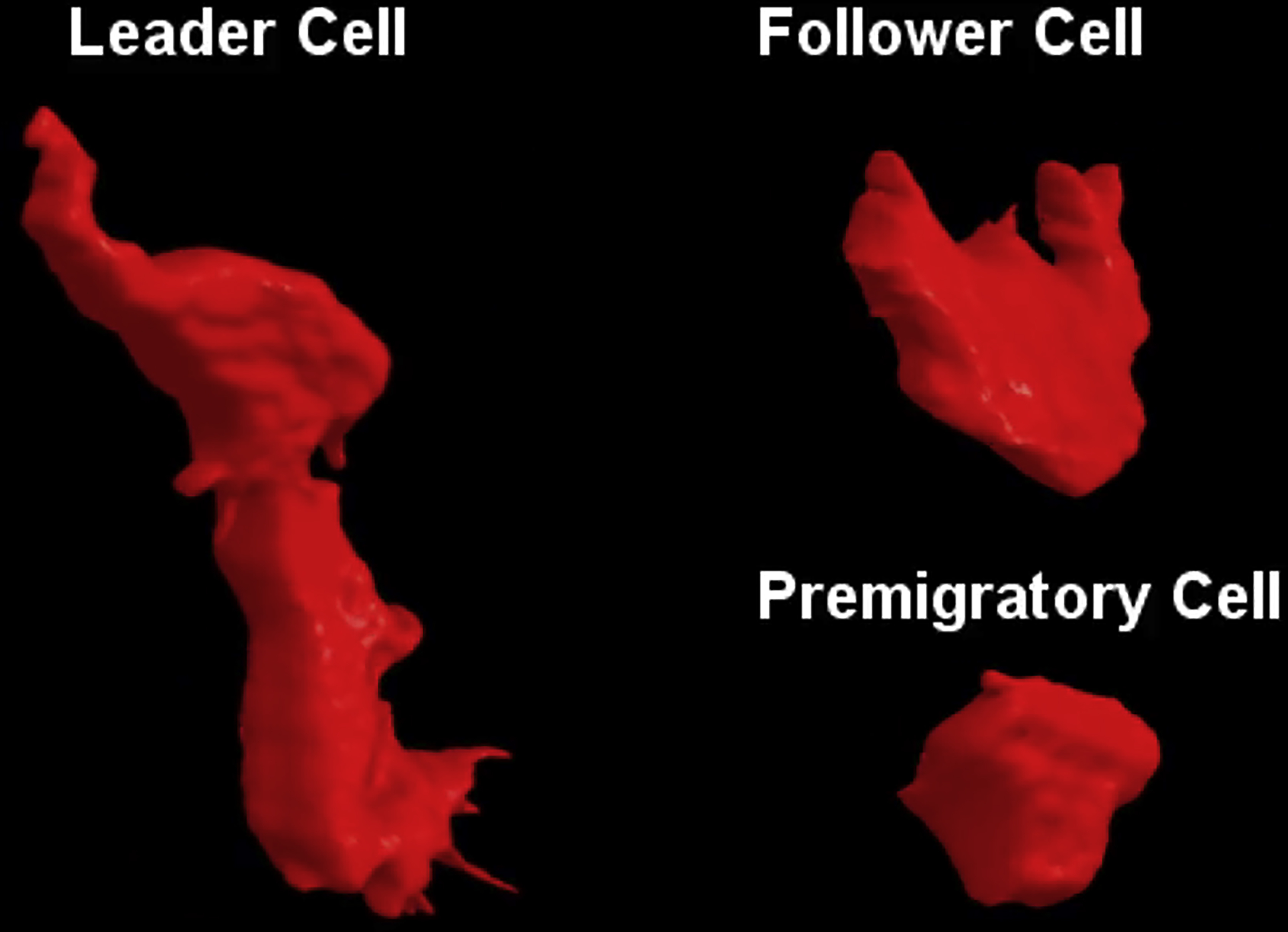

Supplement: Movie S10. Trunk Neural Crest Norphology in Zebrafish, Related to Figure 5 — The movie shows 3D models of leader, follower, and premigratory cells (lateral view, dorsal top, and anterior left, 360° rotation). [file mmc11.jpg]

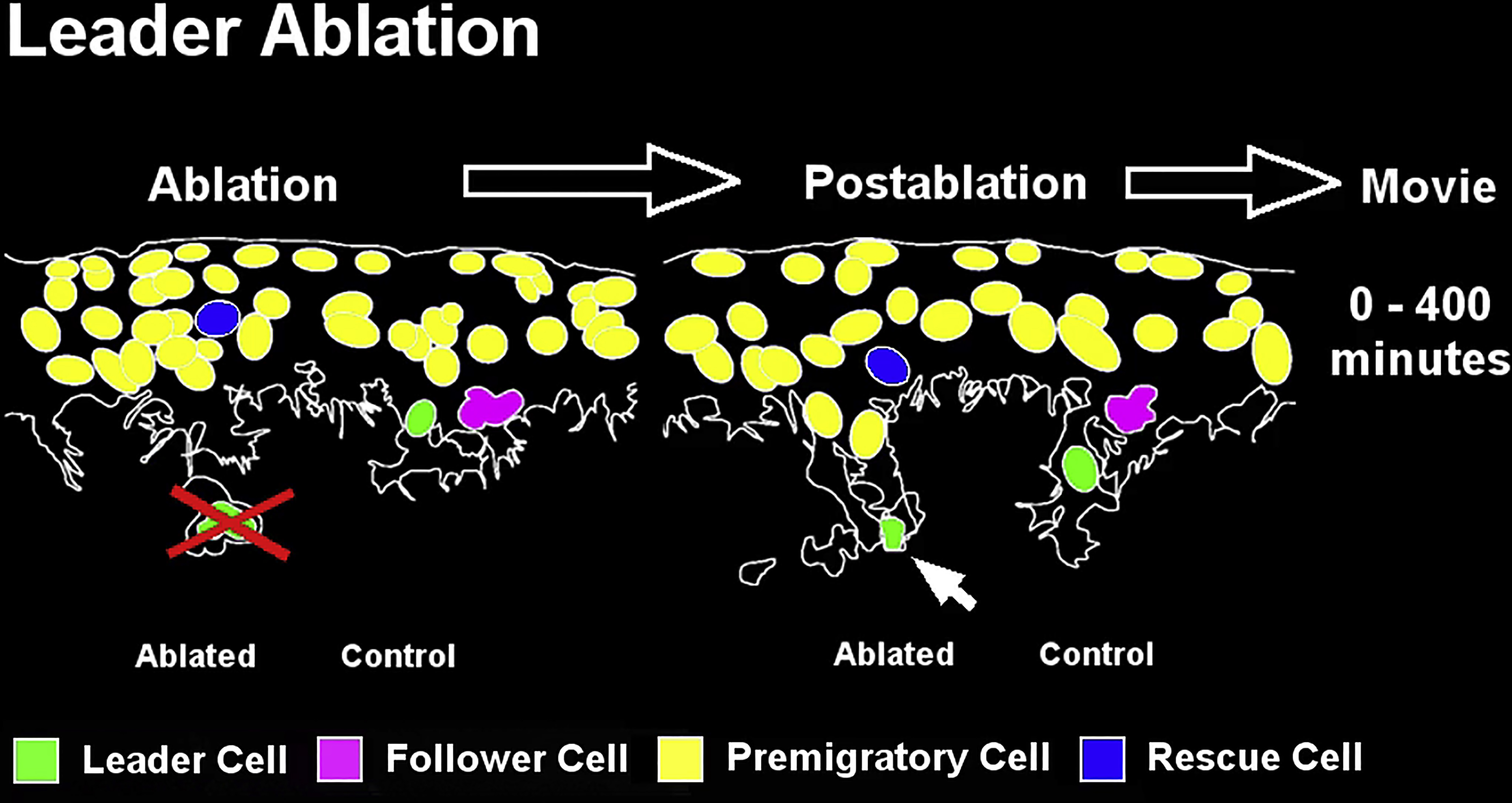

Supplement: Movie S11. Trunk Neural Crest Leader Cell Ablation in Zebrafish, Related to Figure 6 — The movie shows two examples of leader cell laser ablation experiments. The sequence for both examples: cartoon representation of the experiment, followed by the preablation and postablation snapshots and time-lapse movie. The segments 8–9 are shown. The left panel shows a maximal Z projection of fluorescent channels from a Sox10:mG embryo. The right panel shows a maximal Z projection of nuclear fluorescence overlaid by the tracks of leader and follower cells. The images were taken every 5 min (lateral view, dorsal top, and anterior left). [file mmc12.jpg]

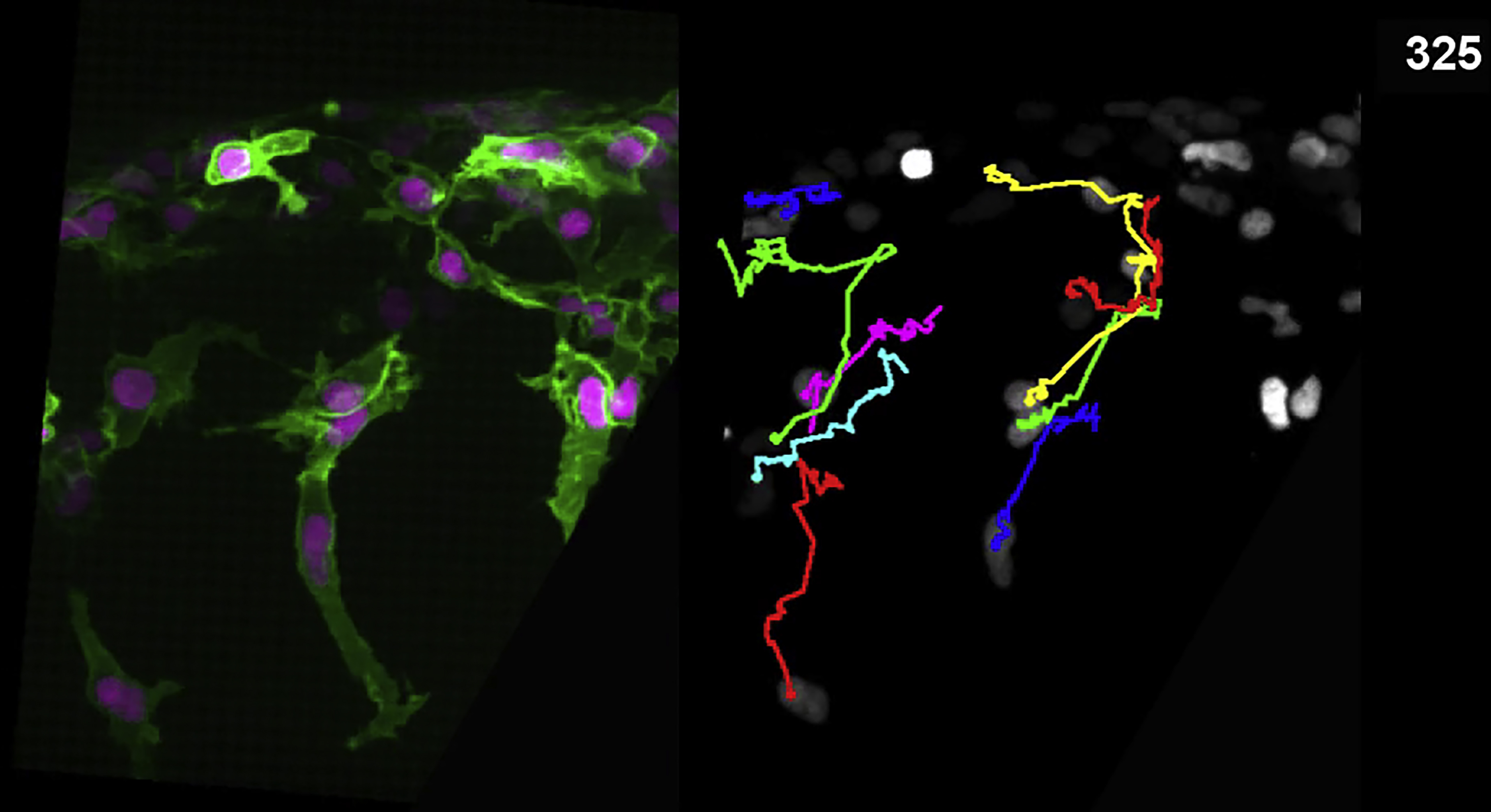

Supplement: Movie S12. Trunk Neural Crest Ablation of Follower Cells: Gap and Follower Ablation in Zebrafish, Related to Figure 7 — The movie shows two examples of follower cell laser ablation generating a gap and one example of follower cell laser ablation that maintains cell contact continuity in the chain. The sequence for every example: cartoon representation of the experiment, followed by the preablation and postablation snapshots, and time-lapse movies. The segments 8–9 are shown. The left panel shows a maximal Z projection of fluorescent channels from a Sox10:mG embryo. The right panel shows a maximal Z projection of nuclear fluorescence overlaid by the tracks of leader and follower cells. The images were taken every 5 min (lateral view, dorsal top, and anterior left). [file mmc13.jpg]
